# Determining factors for compensatory movements of the left arm and shoulder in violin playing

**DOI:** 10.3389/fpsyg.2022.1017039

**Published:** 2023-01-23

**Authors:** Oliver Margulies, Matthias Nübling, William Verheul, Wulf Hildebrandt, Horst Hildebrandt

**Affiliations:** ^1^Music Physiology/Musicians’ and Preventive Medicine Section, Department of Music, Institute for Music Research (IMR), Zurich University of the Arts (ZHdK), Zürich, Switzerland; ^2^Institute for Anatomy and Cell Biology, University of Marburg, Marburg, Germany; ^3^Swiss University Center for Music Physiology, Basel University of the Arts, Basel, Switzerland

**Keywords:** violin ergonomics, 3D motion capture, 2D video analysis, biomechanics, music physiology, musicians’ medicine, prevention

## Abstract

**Introduction:**

Despite a large number of available ergonomic aids and recommendations regarding instrument positioning, violin players at any proficiency level still display a worrying incidence of task-specific complaints of incompletely understood etiology. Compensatory movement patterns of the left upper extremity form an integral part of violin playing. They are highly variable between players but remain understudied despite their relevance for task-specific health problems.

**Methods:**

This study investigated individual position effects of the instrument and pre-existing biomechanical factors likely determining the degree of typical compensatory movements in the left upper extremity: (1) left elbow/upper arm adduction (“Reference Angle α”, deviation from the vertical axis), (2) shoulder elevation (“Coord x”, in mm), and (3) shoulder protraction (“Coord y”, in mm). In a group of healthy music students (*N* = 30, 15 m, 15 f, mean age = 22.5, SD = 2.6), “Reference Angle α” was measured by 3D motion capture analysis. “Coord x” and “Coord y” were assessed and ranked by a synchronized 2D HD video monitoring while performing a pre-defined 16-s tune under laboratory conditions. These three primary outcome variables were compared between four typical, standardized violin positions varying by their sideward orientation (“LatAx-CSP”) and/or inclination (“LoAx-HP”) by 30°, as well as the players’ usual playing position. Selected biomechanical hand parameter data were analyzed as co-factors according to Wagner’s Biomechanical Hand Measurement (BHM).

**Results:**

Mean “Reference Angle α” decreased significantly from 24.84 ± 2.67 to 18.61 ± 3.12° (*p* < 0.001), “Coord x” from 22.54 ± 7.417 to 4.75 ± 3.488 mm (*p* < 0.001), and “Coord y” from 5.66 ± 3.287 to 1.94 ± 1.901) mm (*p* < 0.001) when increasing LatAx-CSP and LoAx-HP by 30°. Concerning the biomechanical co-factors, “Reference Angle α”, “Coord y”, but not “Coord x”, were found to be significantly increased overall, with decreasing passive supination range (*r* = −0.307, *p* = <0.001 for “Passive Supination 250 g/16Ncm”, and *r* = −0.194, *p* = <0.001 for “Coord y”). Compensatory movements were larger during tune sections requiring high positioning of the left hand and when using the small finger.

**Discussion:**

Results may enable to adapt individually suitable instrument positions to minimize strenuous and potentially unhealthy compensation movements of the left upper extremity.

## 1. Background

There is a growing awareness and number of publications on the epidemiology of task-specific health issues in professional musicians in general (Fishbein et al., 1988; Wu, 2007; Vervainioti and Alexopoulos, 2015; Berque et al., 2016) and players of high-stringed instruments specifically. Research dedicated to the latter group reports some of the highest levels of task-specific health problems (Fry, 1986; Dawson, 2001; Berque and Gray, 2002; Aki and Yakut, 2003; Vinci et al., 2015; Kochem and Silva, 2017). Contributing co-factors may be one-sided posture and movement patterns when acquiring the necessary skills (Ericsson et al., 1993; Mornell, 2009; Ranelli et al., 2011; Gembris et al., 2020), but also the realities of professional activity encountered later on when performing and teaching (Spahn et al., 2014; Steinmetz, 2016; Smithson et al., 2017; Rensing et al., 2018; Schemmann et al., 2018; Gembris et al., 2020; Zaza and Farewell, 2001). The prevention of task-specific health problems in musicians has been receiving increased attention, with a growing number of initiatives offering musicians concepts on how to safeguard their health at various stages of their career (Spahn et al., 2001; Hildebrandt and Nübling, 2004; Hildebrandt, 2009, 2017). The scientific basis for a better understanding of ergonomics in violin playing and teaching is gaining grounds (Szende and Nemessury, 1971; Ackerman and Adams, 2004; Wagner, 2005; Wagner, 2012; Rensing et al., 2018; Chi et al., 2020), but co-exists with a wide and contradictory spectrum of long-standing teaching and performing traditions (Flesch, 1978; Galamian, 1983; Rostal, 1993; Rônez-Kubitschek, 2012). Gaining insight into the use of the left upper extremity when playing the violin is a research topic often contributed to in recent years (Blum, 1995a,b; Zaza, 1998; Künzel, 2000; Ackermann and Adams, 2003; Shan and Visentin, 2003; Nyman et al., 2007; Rabuffetti et al., 2007; Wahlström and Fjellman-Wiklund, 2009; Obata and Kinoshita, 2012; Lee et al., 2013; Reynolds et al., 2014; Möller et al., 2018). Nevertheless, only few studies focusing on individual physical predispositions concerning the instrument could be identified (Ackermann and Roger, 2003; Steinmetz et al., 2006; Storm, 2006; Rabuffetti et al., 2007; Seidel et al., 2009). The number of studies dedicated to electromyographic (EMG) measurements of violinists is growing. They examine a broad spectrum of relevant aspects, such as the influence of ergonomics, anthropometrics, and repertoire (Philipson et al., 1990; Cattarello et al., 2017, 2018; Kok et al., 2019; Chi et al., 2020; Mann et al., 2021), the comparison between muscle activation levels in healthy violinists and those reporting task-specific health problems (Spahn et al., 2001; Berque and Gray, 2002; Fjellman-Wiklund et al., 2004; Hildebrandt and Nübling, 2004; Moore et al., 2008; McCray et al., 2016), muscular variability, endurance and fatigue aspects of violin performance (Shan et al., 2004; Wagner, 2005; Gembris et al., 2020; Rousseau et al., 2020). In contrast, research comparing subjectively perceived effort levels and objective data on muscle activation when playing the violin appears to be scarcer (Chan et al., 2000; Hildebrandt et al., 2021). While contributions to the research on anthropometrics of violin playing are available (Ackermann and Adams, 2003; Visentin et al., 2008; Shan et al., 2012; Kelleher et al., 2013; Visentin et al., 2015; Chi et al., 2020), the research of Christoph Wagner on the biomechanics of musicians’ hands remains a cornerstone in this area. It offers the possibility of measuring and understanding intra-individual differences between passive and active mobility ranges attributable to biomechanical co-factors. These in turn are relevant for the performance of an instrument and thus forms an element of this research and paper (see also section 3.4.3 Wagner, 1974, 1977, 2005, 2012; Wilson et al., 1991, 1993; Margulies and Hildebrandt, 2014; Hildebrandt and Margulies, 2018; Nemcova and Hildebrandt, 2018; Hildebrandt et al., 2019; Margulies et al., 2021; Zurich Centre for Musicians’ Hands, 2022).

## 2. Aims, research question, and current status of research

### 2.1. General aims

This research project aims to contribute to the scientific foundation of an individualized ergonomic approach to violin positioning for playing, thereby offering ways of preventing task-specific health problems and delineating solutions for violinists in a rehabilitation or a learning environment.

### 2.2. Research question

For this publication, the research focuses on how an instrument’s given position affects the degree of compensation movements of the left upper extremity and how individual biomechanical properties of the left upper extremity factor into the pattern of compensation movements.

### 2.3. Current status of own research

**Figure 1:**
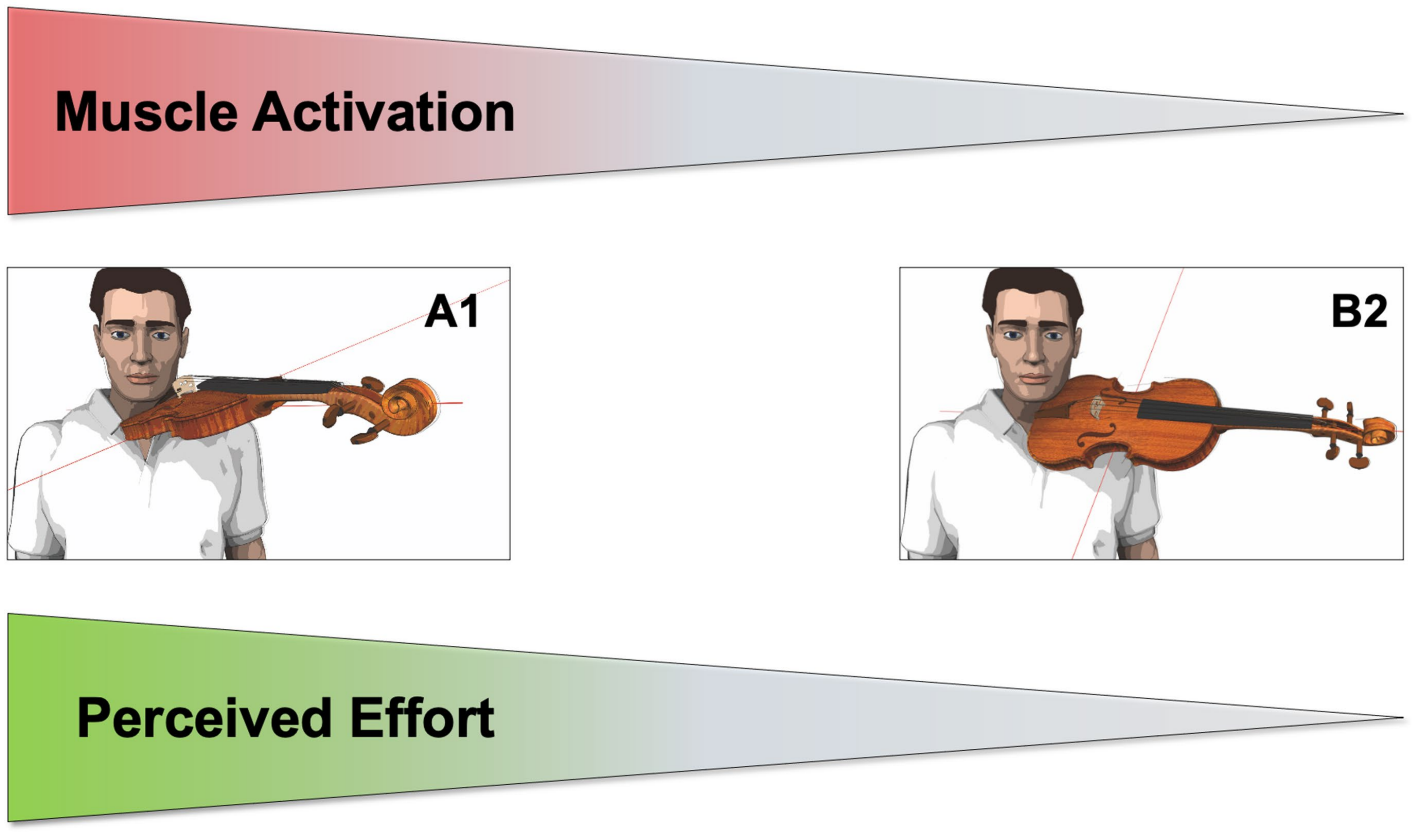
Schematic illustration of the influence of an instrument’s sidewards orientation and inclination on muscle activation and perceived effort.

Summarizing recently published findings for muscle activation (EMG) and subjectively perceived effort (Borg-Scale) in violin players, it was shown that muscle activation and perceived effort within the violinists’ left arm increased when the sideward orientation and inclination of the instrument in playing were smaller in angle relative to the respective plane (see Figure 1; Hildebrandt et al., 2021).

With a 30° decrease in the instrument’s sideward orientation (i.e., the instrument’s longitudinal axis nearer the central sagittal plane and more in front of the player) and the instrument’s inclination (i.e., the instrument’s lateral axis nearer the horizontal plane and flatter), mean values of overall muscle activation and subjectively perceived effort in the violinist’s left arm increased highly significantly and independently. Among the four muscles measured due to their involvement in movements when playing the violin (i.e., *M*. *pectoralis* major, *M*. *biceps brachii*, *M*. *extensor carpi ulnaris*, and *M*. *extensor digitorum communis*), effects in muscle activation (EMG) were especially observable for the pectoralis major muscle which is mainly involved in moving the upperarm forward relative to the trunk. This can be considered a typical compensation movement in violin performance, the analysis of which is at the core of this paper.

### 2.4. Specific aims linked to this paper

This paper aims to describe:

(a) The degree of compensation movement of a player’s left upper extremity when playing the violin in four standardized instrument positions and a player’s normally used instrument position (see Table 1 below). The degree of compensation movement becomes observable, (i) in the player’s left elbow’s movements relative to the central sagittal plane, and (ii) in the movements of the player’s left acromion in a combined upward and forward movement (elevation and protraction).

**Table 1:** Four standardized violin positions with orientation points and the free position.

Position	Description of violin position
**A1** 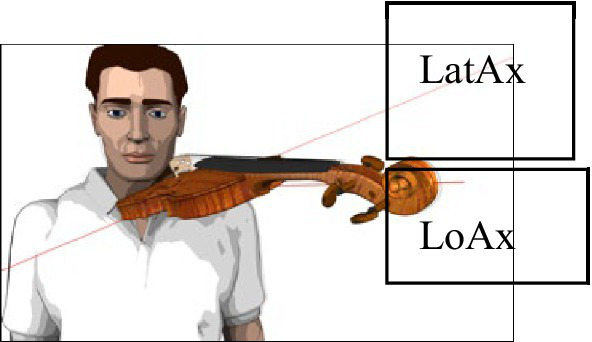	The longitudinal central instrument axis (LoAx) is at a 20° angle to the player’s central sagittal plane (CSP) and points towards the left lamina of the thyroid cartilage (vertical *via* the clavicular insertion of the left sternocleidomastoid muscle). The lateral instrument axis (LatAx) deviates 20° from the player’s horizontal plane (HP).
**A2** 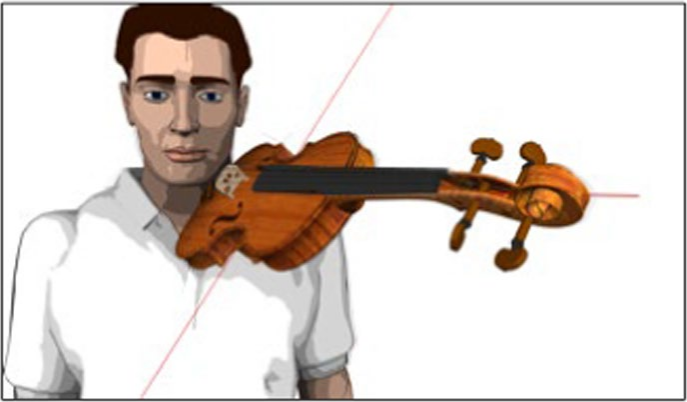	LoAx is identical to A1 (20° deviation from player’s CSP), however LatAx deviates 50° instead of 20° from HP.
**B1** 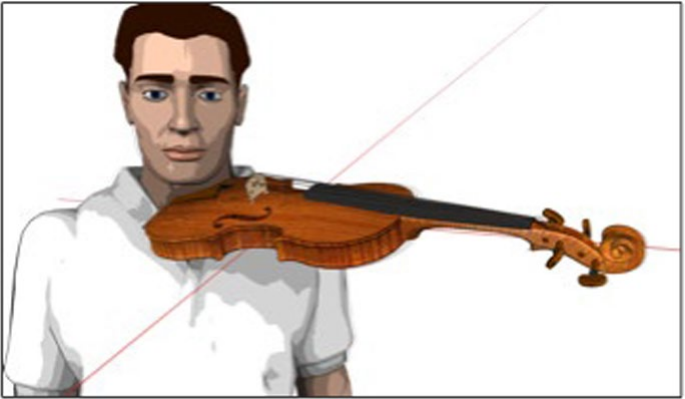	LatAx is identical to A1 (20° deviation from player’s HP), however, LoAx deviates 50° instead of 20° from the player’s CSP (with the LoAx extension running to the clavicular insertion of the right).
**B2** 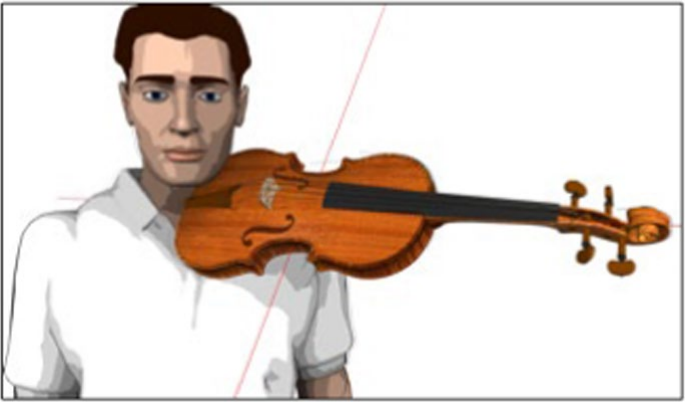	LoAx is identical to B1 (50° from player’s CSP), however LatAx deviates 50° instead of 20° from player’s HP.
**Free**	Free playing position with normally used personal ergonomic adaptations to the violin as used in real-life setting by study participant.

(b) How specific biomechanical parameters affect the other target parameters included in this study (see section 3.4 below).

### 2.5. Hypotheses

#### 2.5.1. Hypotheses for compensation movements in left elbow and acromion

For compensation movements in the left elbow and acromion, the following hypotheses were formulated:

The degree of compensation movement of a violinist’s left elbow expressed as “Reference Angle α” (see section 3.4.1), “Coord x” for shoulder protraction, and “Coord y” for shoulder elevation seen in the left acromion (see section 3.4.2) will increase, (1) the more the longitudinal axis (LoAx) of the instrument points toward the front, i.e., approaches the player’s central sagittal plane (CSP) by reducing the angle between LoAx and CSP from 50° to 20° (see Table 1: violin positions B vs. A), and (2) the more horizontal the lateral axis (LatAx) of the instrument approaches the player’s horizontal plane (HP) by reducing the angle between LatAx and HP from 50° to 20° (see Table 1: violin positions 2 vs. 1).

#### 2.5.2. Sub-hypotheses for biomechanics

For biomechanics, the following two sub-hypotheses were formulated:

Sub-hypothesis 1: The lower the passive supination ability (see section 3.4.3), the higher the degree of compensation movement in the violinist’s left arm when playing.

Sub-hypothesis 2: The shorter the length of the little finger in comparison with the length of the middle finger (see section 3.4.3) and the lower the passive thumb spreading ability (see section 3.4.3), the higher the degree of compensation movement in the violinist’s left arm when playing.

## 3. Materials and methods

### 3.1. Study design

The study was designed as a cross-sectional study. It included 30 healthy violinists (15 male and 15 female) in professional formation (BA and MA studies). Study participants were recruited from Swiss music universities and the Vorarlberg State Conservatory, Feldkirch, Austria. The mean age of the study population was 22.5 years (Min. 18, Max. 29 years, SD = 2.6). The study was approved by the Canton of Zürich Ethics Committee (project no. KEK-ZH-Nr. 2014–0008). Study participants gave their informed consent prior to participation.

### 3.2. Measurement steps

**Figure 2:**
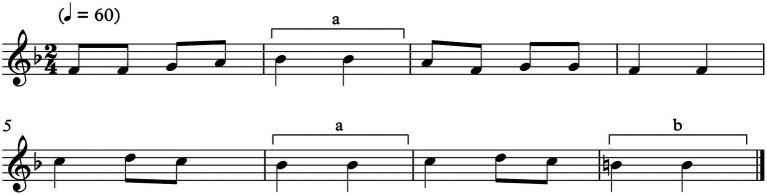
16-s tune used for measurements.

**Figure 3:**
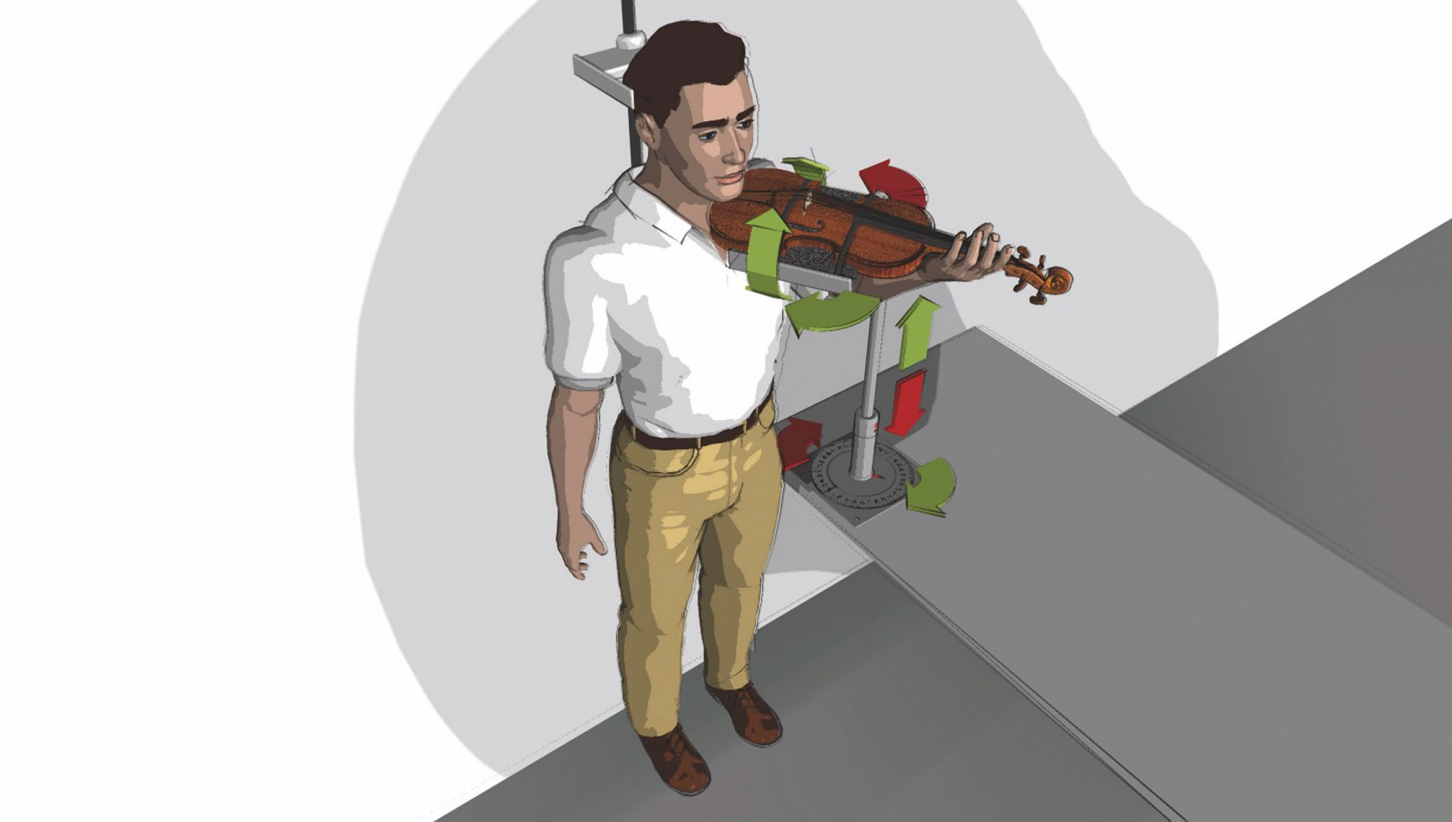
Schematic illustration of instrument fitting device in experiment set-up.

(a) Study participants were asked to play a pre-defined 16-s tune (Figure 2) during ongoing comparative measurements of compensation movements. The tune was played in four randomized and standardized violin positions of a laboratory instrument (see Table 1 and Technical Prerequisites below).

(b) The tune was measured identically, but with study participants playing on their own instrument and with their normally used ergonomic equipment, thereby supporting the weight of the instrument as usual but remaining in the standardized body position as required for the previously measured violin positions A1 through B2 (Table 1 and Figure 3).

The tune included a total of three note sequences with a low (“a”) vs. high (“b”) small finger (digitus minimus, see Figure 2) in both the sixth and second hand position. Finger placement “a” represents a more rounded finger closer to the ring finger, and finger placement “b” a more extended finger abducted from the ring finger. Both fourth-finger positions are frequently required in violin performance.

(c) Biomechanical data was collected in the hand laboratory available to the research team (see section 3.4.3).

### 3.3. Technical prerequisites

#### 3.3.1. Standardization of violin positions

Ensuring measurements under standardized conditions (see Table 1) required the development of a specific device (schematically depicted in Figure 3). This device meets the following requirements: (1) three-dimensional fitting of the laboratory violin to the player with precisely reproducible heights and angles, (2) minimization of excess holding work in head, neck, shoulder, arm, and hand while playing a laboratory instrument without ergonomic aids, and (3) exclusion of confounding variables such as individual playing and postural habits as well as ergonomic equipment (see also measurement procedures below).

This methodology allowed for intraindividual comparison between all violin positions tested (see Table 1). The standardized instrument positions were defined by estimates based on a range of recommendations of internationally renowned players’ and teachers’ instrument positions and considered typical in teaching traditions of the last few centuries (Flesch, 1978; Galamian, 1983; Rostal, 1993; Rônez-Kubitschek, 2012).

The laboratory instrument had the following dimensions: body length = 354 mm, vibrating string length = 328 mm. These dimensions correspond to a standard, full-size violin commonly used. For measurements, it was fitted without ergonomic equipment (i.e., chin-rest or shoulder pad). String tension was specified by tuning the instrument to concert pitch 442 Hz.

### 3.4. Target parameters

#### 3.4.1. 3D motion capture data for elbow compensation movements (Reference Angle α)

Data was acquired with an opto-electronic 3D movement analysis device, Model MCU 200, company Laitronic (Innsbruck, Austria). High-resolution recordings of infrared signals permit differentiated statements and documentation of the relative marker position changes in comparison with each other (Bannach et al., 2009). While the 16-s tune was played with a metronome set to 60 bpm, the system recorded changes in Reference Angle α values at a frame rate of 200.00 Hz. Throughout all measurements, the camera unit was kept at a constant height (160 cm), distance (190 cm) and angle (45°) relative to the study participant and experiment set-up.

**Figure 4:**
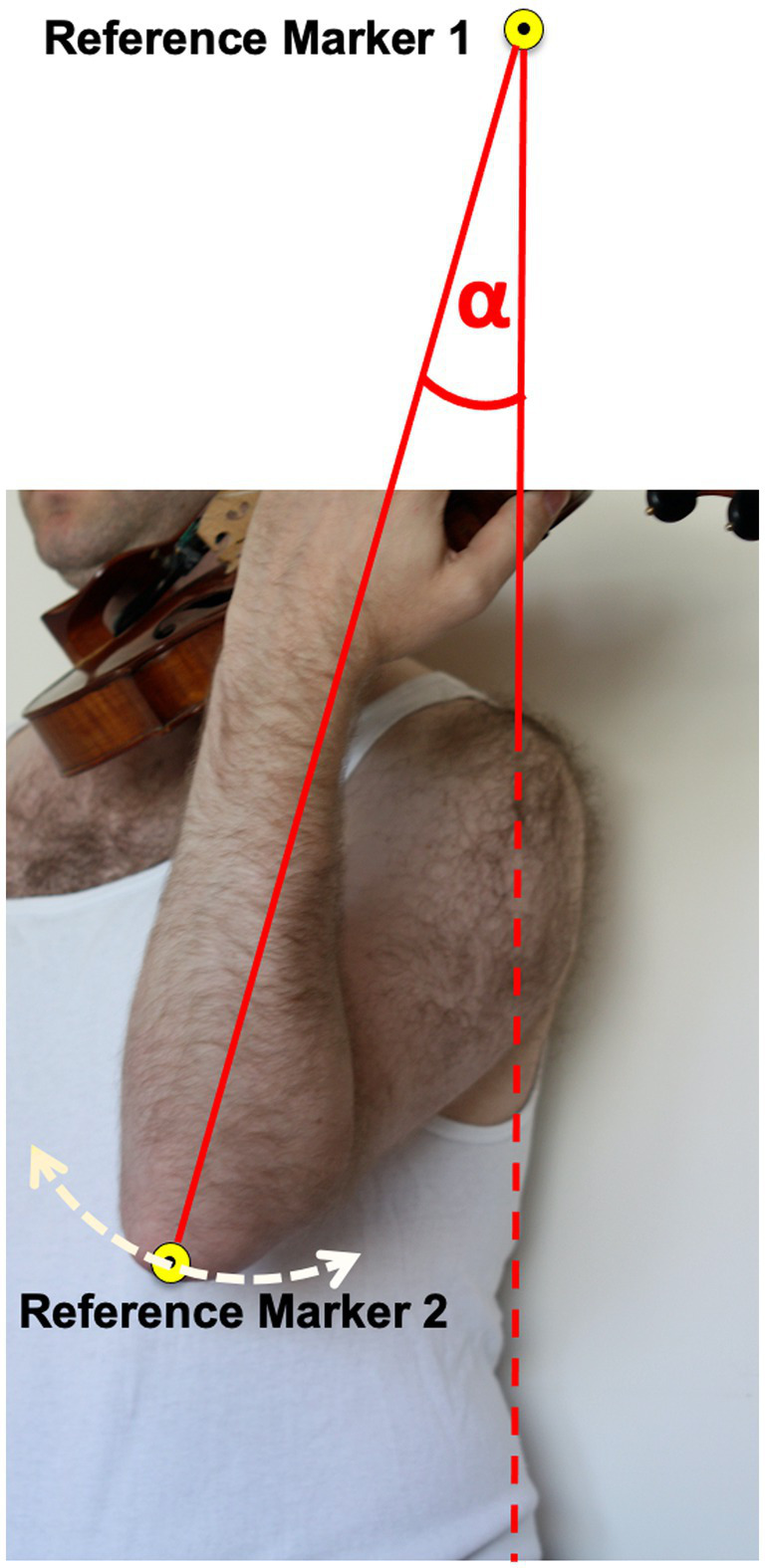
Schematic illustration of Reference Markers 1 and 2, and Reference Angle α.

Motion capture data were collected in the four standardized instrument positions, and the violinist’s own normally used position with his/her own instrument (Table 1). Two markers define Reference Angle α:

1. Reference Marker 1 was positioned at a standardized height of 212 cm on the back wall of the device holding the instrument. It was aligned with the study participant’s left acromion by a plumb hanging from Reference Marker 1.
2. Reference Marker 2 was positioned on the study participant’s left olecranon (see Figure 4).

#### 3.4.2. 2D motion capture data for acromion compensation movements (“Coord x” and “Coord y”)

To record the shoulder’s motion, a Panasonic HDC-HS300 video camera mounted on a tripod was used. For each study participant, the camera was positioned the following way: (i) the camera lens at the height of the left acromion, (ii) the longitudinal axis of the camera in-line with the acromion’s transversal axis, and (iii) at a standardized distance between the study participants’ acromion and the camera lens. Before filming shoulder motion, an “X” was marked on the study participant’s skin for reference. Data for acromion motion were collected at the same time as data for Reference Angle α during the 16-s tune.

**Figure 5:**
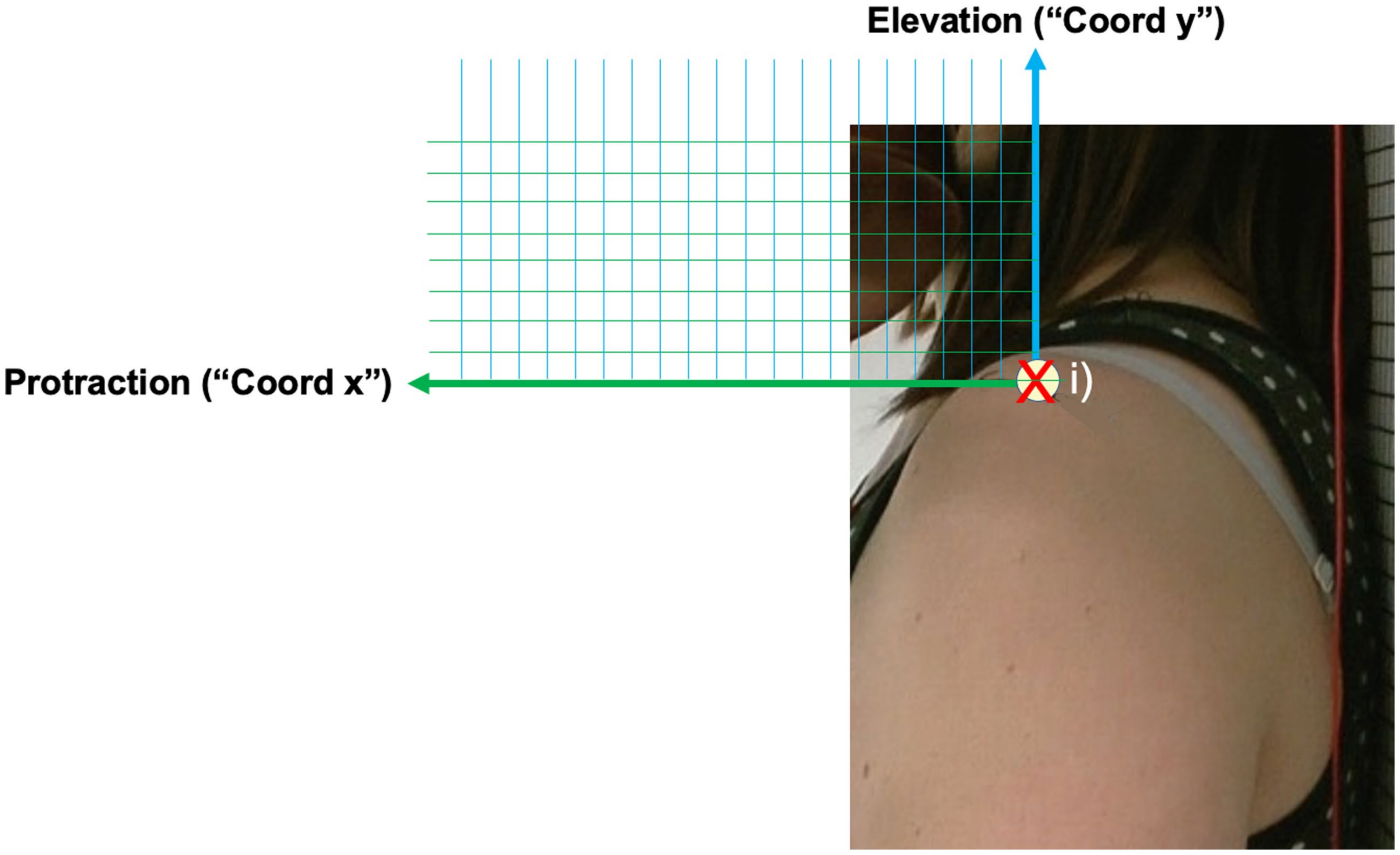
Schematic illustration of acromion motion and measuring grid.

The target parameters for this data set were expressed as follows: (a) “Coord x” for the forward movement of the shoulder (protraction) and (b) “Coord y” for the upward movement of the shoulder (elevation). Data for “Coord x” and “Coord y” was collected at four points in time for each with a running metronome set to 60 bpm:

- Five seconds before playing the tune, the left arm hanging in neutral position next to the body (see Figure 5).
- At the beginning of the first note of the tune (see Figure 2).
- At the beginning of bars 2 and 6 of the tune, where the regular fourth finger is played (see brackets marked “a”, in Figure 2).
- At the beginning of the tune’s last bar, where the extended fourth finger is played (see brackets marked “b”, in Figure 2).

Shoulder data was collected post-measurement by replaying the video sequences on a 13-inch Apple® MacBook Air with the Software QuickTime Player, Version 10.5, in the full-screen setting with a transparent millimeter grid over the computer’s screen. Data for “Coord x” and “Coord y” were generated by tracking the acromion’s movements and marking the relevant points in time on the millimeter grid. This resulted in a data pair (“Coord x”/ “Coord y”) expressing the degree of protraction and elevation per observed point in time. Data was then transferred onto a spreadsheet for further analysis.

#### 3.4.3. Biomechanical data for hand parameters

**Figure 6:**
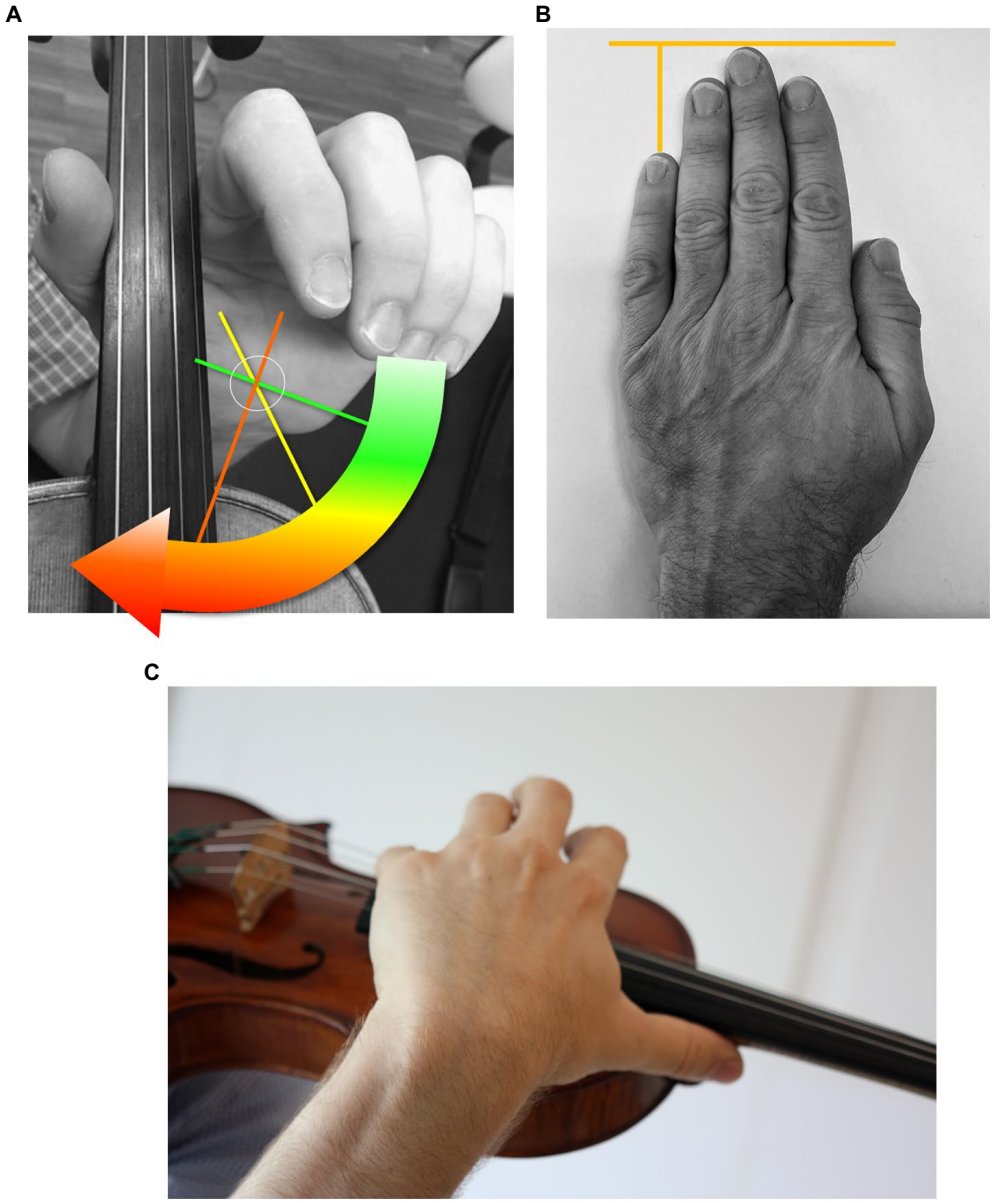
**(A)** Passive supination range. **(B)** Difference in finger length between third and fifth fingers. **(C)** Thumb spreading in high positions on the violin.

For biomechanical data, the following target parameters were defined:

1. Passive supination ability of the left lower arm at torque levels 16 Ncm (or 250 g weight) and 30 Ncm (or 500 g weight): Indicates effort or ease of the hand reaching basic positions on the violin. Measurements result in an angle degree describing the deviation of the left forearm, hand, and wrist from the neutral position (Figure 6A).
2. Difference in finger length between the left hand’s third and fifth fingers: Indicates the fifth finger’s anatomical position (i.e., shortness relative to the middle finger (Figure 6B). Measurements result in a millimeter value.
3. Passive thumb spreading ability: Indicates the deviation of the thumb relative to the second metacarpal bone (Figure 6C). Measurements result in an angle degree value.

Passive movement range generated with an external torque is particularly relevant, as differences are more evident than in the assessment of *active* movement range (Wagner, 1977). Routine clinical examinations often record values for *active* movement range, but significant deficits in *passive* movement range with little torque remain unobserved. Such individual “movement brakes” in joint structures and tissue properties may provoke players to “force” themselves into a required playing position and provoke additional compensation movements. To measure biomechanical data, and particularly the passive movement ranges, the research team used the original laboratory apparatuses developed by Christoph Wagner for the Biomechanical Hand Measurement (BHM; Wagner, 2005; Wagner, 2012). The examination of the hand and arm only uses non-invasive, mechanical measurement methods. As a result of the measurements, an objective and differentiated image of individual, instrument-specific possibilities and limitations of the musician’s hand against the background of instrument-specific comparison groups are obtained. These results then permit the comparison of individual data with the existing data pool generated over more than five decades of research from fellow professionals of a given instrument.

Finger Length Difference 3–5 is measured using a mechanical gauge with the hand in a standardized position (see Figure 6B above). For passive supination and passive thumb spreading [see Figures 6A,C above], a subject first assumes a standardized body position (standing for passive supination and sitting for passive thumb spreading). Then, the hand and arm positions of the subject are equally standardized for measurement in the apparatuses. Subjects are then instructed to remain as relaxed as possible without actively contributing to the joint movement measured. Measurements for passive mobility of the parameter are then carried out by the pre-defined torque generated by weights hanging from the apparatuses. The first moment of the hand’s or arm’s inner resistance against the pre-defined torque results in an angle degree. The average of three tests is then documented as the individual’s measure for passive mobility. For further reference, please refer to www.zzm.ch.

### 3.5. Study participant positioning

The participants were asked to position themselves on the instrument fitting device’s platform (see Figure 3). They stood upright, head position looking straight ahead at the music stand on which the tune was placed. Individually adjustable stabilizers were positioned on both sides of the study participants’ head to ensure that no additional strain on the neck and shoulder muscles would occur during measurement. They were also instructed to choose a shoulder position they considered as relaxed as possible before going into playing position. The positions of feet and knees were defined, marked, and positioned so they could stand comfortably throughout the measurement phases. During all measurements, study participants were asked to remain relaxed but in the same body position to allow for accurate intraindividual data comparisons. The body position was documented with two video cameras (frontal and from the side).

### 3.6. Measurement procedure

All tests were carried out in a randomized order. Measurements for each of the four violin positions A1 to B2 (see Table 1) and the position chosen by the participant for the own instrument (Free) included the following steps:

- Within a 10-s countdown, the participant went into playing position with the left hand.
- The fingers were positioned over the notes specified by the tune on the lowest string in sixth position.
- The participant played the tune (Figure 2) without a bow and then let the left hand sink back down again into the relaxed starting position.
- During a break, the study participant relaxed the hand and arm (movements, self-massage, tapping, and shaking were avoided) (Hildebrandt et al., 2021).
- The same procedure was applied when playing the tune in second position and a second time in the sixth position.

To rule out fatigue effects on compensation movement patterns attributable to playing in the more demanding sixth position for a prolonged time, alternating between the sixth and the second playing position was invariably applied for all violin positions tested. Means of the sixth and second playing positions were used to compare the five violin positions tested.

### 3.7. Statistical procedures

For 3D motion capture analysis describing elbow and upper arm compensation movement, measurements of the 30 study participants playing 15 repeats of the tune yielded a total of 450 observations for “Reference Angle α” For 2D motion capture data describing shoulder motion, measurements yielded a total of 450 observations per point in time observed for “Coord x” and “Coord y” each. Biomechanical data collected yielded a total data set of 30 observations per parameter (one measurement per person).

For 3D motion capture data, mean values for “Reference Angle α” were calculated by hand position for each of the five pre-defined violin positions (A1, B1, A2, B2, and Free) and the overall mean value for all 450 data points. For 2D data, mean values for “Coord x” and “Coord y” were calculated by hand position for all four points in time and all violin positions tested. For biomechanical data, mean values were calculated for each of the parameters examined.

The separate contribution of the five violin positions under test to elbow/upper arm compensation (3D), shoulder motion (2D) and biomechanical data variability was assessed by multiple linear regression analysis, choosing position A1 as the reference category, describing deviations from A1 for the other four positions.

A sub-analysis for 3D data was carried out for the entire duration of the 16-s tune as well as the time segments where the normal and high fourth (small) finger was used in both second and sixth hand position (Figure 2, brackets “a” and “b”). Differences in overall 3D and 2D values between the single instrument and finger positions were assessed for all combinations by a *post hoc* multiple comparison of means (Scheffé), with significance levels reported as *p < 0.05, **p < 0.01, and ***p < 0.001. Correlations (Pearson’s r) were calculated between the following parameters: (a) shoulder motion data (2D) and elbow/upper arm compensation movement (3D), (b) biomechanical parameters and elbow/upper arm compensation movement (3D) and (c) shoulder motion data (2D) and biomechanical parameters, with significance levels reported as *p < 0.05, **p < 0.01, and ***p < 0.001.

All data analysis was carried out using the SPSS 20 statistics program for Windows (IBM SPSS, Armonk, NY, United States).

## 4. Results

### 4.1. Results for the entire 16-s tune by instrument position

#### 4.1.1. Elbow/upper arm compensation movement by instrument position (Reference Angle ɑ)

**Figure 7:**
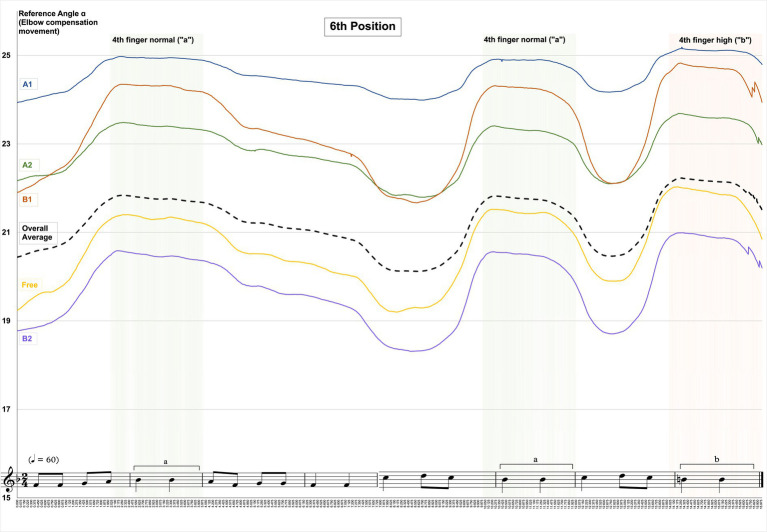
Changes in Reference Angle α by instrument position and overall mean when playing in sixth hand position.

**Figure 8:**
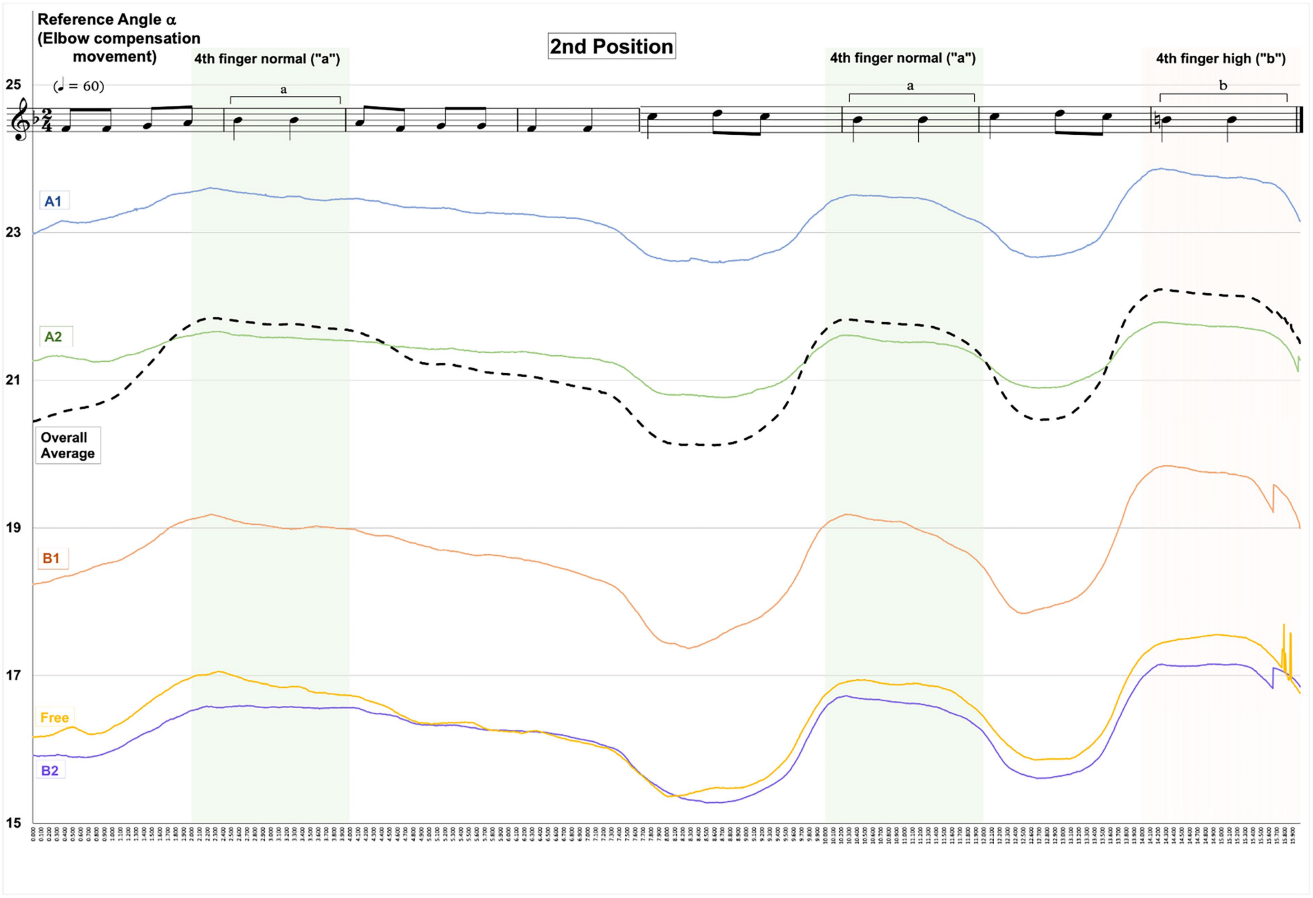
Changes in Reference Angle α by instrument position and overall mean when playing in second hand position.

A comparison between the five tested violin positions (see Table 1) shows the highest degrees of elbow/upper arm compensation for violin position A1 compared to all other positions and the lowest values for violin position B2, the overall mean of aggregated data (corresponding to the dashed line in Figures 7, 8) is 3.5 points lower than A1 (see Figures 7, 8 and Supplementary Table S1).

**Figure 9:**
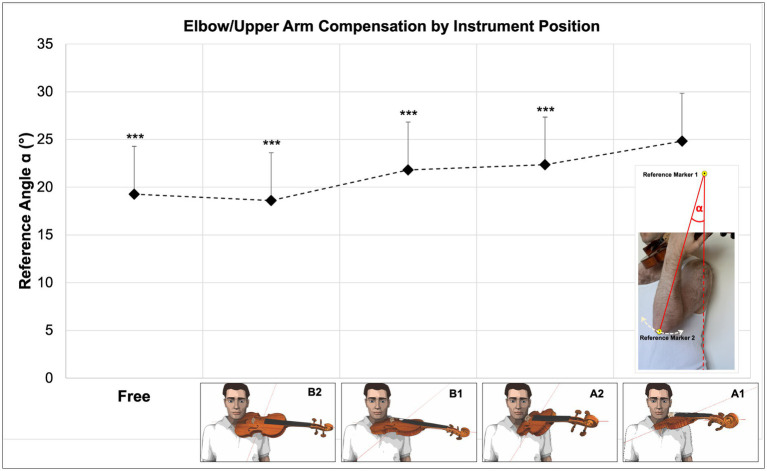
Degree of elbow/upper arm compensation movement expressed as Reference Angle α_ by instrument position. Differences between violin position A1 and all other instrument positions under test are highly significant (*p* < 0.001).

Multiple regression analysis for “Reference Angle α” by instrument position shows highly significant differences between violin position A1 and all other instrument positions (*N* = 90, *p* < 0.001, r^2^ = 0.353). R^2^ for the entire model explains 35% variance of elbow/upper arm compensation movement considering all hand positions for a given instrument position (see Figure 9 below and detailed regression analysis in Supplementary Table S2).

A *post hoc* multiple comparison of means (Scheffé) shows that, for all hand positions united (*N* = 450), mean values between single instrument positions differ highly significantly between each other (*p* < 0.001), except A2 to B1 and B2 to Free (*p* < 0.844 and *p* < 0.707, therefore non-significant). For further details, refer to Supplementary Table S3.

#### 4.1.2. Shoulder compensation movements by instrument position (“Coord x”/“Coord y”)

**Figure 10:**
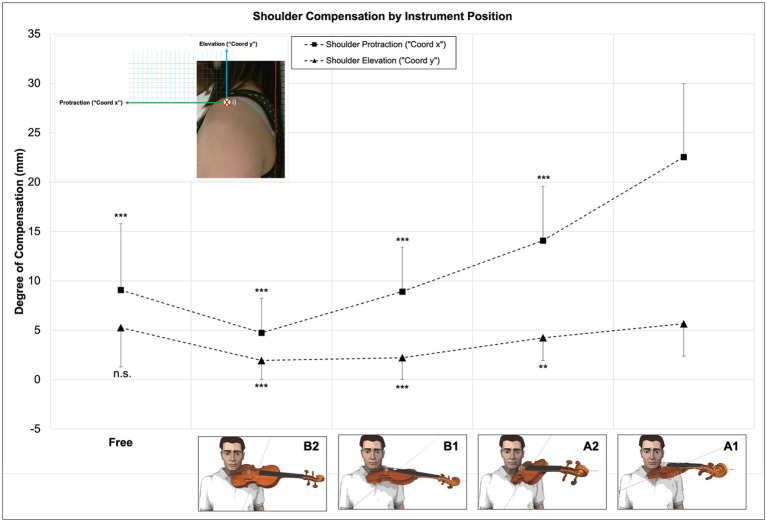
Degree of shoulder compensation movement expressed as “Coord x” and “Coord y” by instrument position. Differences between violin position A1 and all other instrument positions under test highly significant (*p* < 0.001), except for A2 in “Coord y” (*p* < 0.01) and Free in “Coord y” (n.s.).

For shoulder motion data in both x-and y-direction, the highest values are observed for instrument position A1 and the lowest for B2. The gradation of values follows the same pattern for both shoulder movement directions. Values for “Coord x” (protraction) are higher than for “Coord y” (elevation). Instrument Position Free shows values comparable with instrument position B1 for protraction and A1 for elevation. Overall values uniting all instrument and hand positions show higher values for protraction than for elevation (see Figure 10 above and Supplementary Table S4).

Multiple regression analysis for “Coord x” (shoulder protraction) for the entire model, including all instrument and hand positions, shows highly significant differences between instrument position A1 and all other instrument positions (*N* = 90, *p* < 0.001, r^2^ = 0.536), thereby explaining 53% of shoulder protraction movement variance when considering all instrument and hand positions.

Multiple regression analysis for “Coord y” (shoulder elevation) for the entire model, including all instrument and hand positions, shows highly significant differences between instrument position A1 and positions B1 (*N* = 90, *p* < 0.001, r^2^ = 0.226) and B2 (*N* = 90, *p* < 0.001, r^2^ = 0.226). Differences between instrument position A1 and position A2 reach the next-highest significance levels (*N* = 90, *p* < 0.01, r^2^ = 0.226), and differences between instrument position A1 and position Free are non-significant (*N* = 90, *p* = 0.360, r^2^ = 0.226). R^2^ for the entire model is 0.226, thereby explaining between 22% of shoulder elevation movement variance when considering all instrument and hand positions (see Figure 10, above and detailed regression analysis in Supplementary Table S5).

A *post hoc* multiple comparison of means (Scheffé) shows that, for all hand positions united (*N* = 449) and shoulder protraction (“Coord x”) mean values between single instrument positions differ highly significantly between each other (*p* < 0.001), except between instrument position B1 and Free (non-significant). The same *post hoc* test for shoulder elevation (“Coord y”) shows highly significant differences in 12 out of 20 position comparisons (*p* < 0.001), two significant differences between instrument positions A1 and A2 (*p* < 0.05), and six non-significant differences between A1 and Free, A2 and Free as well as B1 and B2. For further details, refer to Supplementary Table S6.

Based on data presented in sections 4.1.1 and 4.1.2, the main hypothesis regarding instrument position effects on the degree of elbow and upper arm compensation (see section 2.5.1 above) is confirmed. For shoulder motion data, the main hypothesis is confirmed for shoulder protraction (“Coord x”) and partially confirmed for shoulder elevation (“Coord y”).

#### 4.1.3. Results for biomechanical data

For biomechanical parameters investigated, Table 2 offers an overview of the data collected.

**Table 2:** Descriptive statistics for biomechanical data (*N* = 30 per parameter).

Biomechanical parameter	*N*	Mean	Min.	Max.	*SD*
Finger Length Difference 3-5	30	36.98	28.5	46.0	3.690
Passive Supination 250 g (16 Ncm torque)	30	51.47	5.0	106.0	34.516
Passive Supination 500 g (32 Ncm torque)	30	84.48	17.0	122.0	23.345
Passive Thumb Abduction 250 g (25 Ncm torque)	30	50.60	39.0	65.0	7.206

The linear relationship between the biomechanical parameters and “Reference Angle α” for the entire duration of the tune was assessed by computing Pearson’s *r* correlation coefficient for all five instrument positions singly and aggregated, all hand positions united, and all three time points under test.

The same linear relationship was assessed for the biomechanical parameters and shoulder protraction (“Coord x”) as well as shoulder elevation (“Coord y”) for the entire duration of the tune.

For the correlations between biomechanical parameters “Passive Supination 250 g” as well as “Passive Supination 500 g” and “Reference Angle α” for all instrument positions under test, including data for all positions aggregated (“Overall”), data shows highly significant negative correlations. The only exception to this rule is observed for “Passive Supination 250 g” for instrument position “Free”, where significance levels reach the lowest level (*p* < 0.05; see Table 3).

**Table 3:** Correlation analysis between biomechanical data and Reference Angle α as well as data for shoulder elevation data *p < 0.05, **p < 0.01, and ***p < 0.001.

Instrument position	*N*	Biomechanical parameter	Reference Angle ɑ		Shoulder protraction (“Coord x”)		Shoulder elevation (“Coord y”)	
			*r*	*p*	*r*	*p*	*r*	*p*
A1	90	Passive Supination 250 g	−0.469	<0.001 (***)	0.029	0.783	−0.320	<0.05 (*)
	90	Passive Supination 500 g	−0.490	<0.001 (***)	0.040	0.710	−0.268	<0.05 (*)
A2	90	Passive Supination 250 g	−0.525	<0.001 (***)	0.020	0.850	−0.287	<0.01 (**)
	90	Passive Supination 500 g	−0.529	<0.001 (***)	−0.060	0.573	−0.206	0.051
B1	90	Passive Supination 250 g	−0.365	<0.001 (***)	0.076	0.474	−0.302	<0.01 (**)
	90	Passive Supination 500 g	−0.349	<0.01 (**)	0.022	0.838	−0.060	0.573
B2	90	Passive Supination 250 g	−0.384	<0.001 (***)	0.112	0.294	−0.326	<0.01 (**)
	90	Passive Supination 500 g	−0.400	<0.001 (***)	0.090	0.398	−0.154	0.147
Free	90	Passive Supination 250 g	−0.257	<0.05 (*)	−0.027	0.803	−0.032	0.765
	90	Passive Supination 500 g	−0.313	<0.01 (**)	−0.123	0.249	0.144	0.175
Overall	450	Passive Supination 250 g	−0.307	<0.001 (***)	0.021	0.655	−0.194	<0.001 (***)
	450	Passive Supination 500 g	−0.321	<0.001 (***)	−0.011	0.821	−0.075	0.112

For shoulder elevation (“Coord y”), data shows negative correlations for “Passive Supination 250 g” except for instrument position Free, and negative correlations for “Passive Supination 500 g” for instrument position A1 (*r* = −0.268, *p* < 0.05).

For the biomechanical parameter “Passive Thumb Abduction,” data shows positive correlations for instrument positions A1 (*r = 0*.270, *p* < 0.05), A2 (*r = 0*.255, *p* < 0.05), B2 (r = 0.226, *p* < 0.05) and Overall (r = 0.143, *p* < 0.01). For instrument positions B1 and Free, correlations are non-significant. For biomechanical parameter “Finger Length Difference 3-5”, data shows no significant correlations. For detailed regression analysis results, please refer to Supplementary Table S7.

The sub-hypotheses focusing on the contribution of biomechanical parameters to the target parameters elbow/upper arm and shoulder compensation movement (see section 2.5.2) were confirmed for “Passive Supination”, not confirmed for “Passive Thumb Abduction” (positive instead of negative correlation) and not confirmed for “Finger Length Difference 3-5” (non-significant correlation).

### 4.2. Results for the entire 16-s tune by hand and instrument position

#### 4.2.1. Influence of sixth and second hand position on elbow/upper arm compensation movement

The following Figures 7, 8 show the patterns and degrees of elbow/upper arm compensation movements expressed as “Reference Angle α” when playing the entire 16-s tune in each of the instrument positions under test, as well as highlighted time sections for the normal fourth fingers (green sections) and high fourth finger (red section).

Changes in values for “Reference Angle α” follow a similar pattern for all instrument and hand positions with a characteristic increase during the time the fourth fingers are played. Values for “Reference Angle α” are at higher levels for sixth position than for second position, and the gradation of values for “Reference Angle α” shows the same pattern, in that instrument position A1 yields the highest, and B2 the lowest levels. However, for sixth hand position, instrument position B1 reaches higher levels than A2, whereas for second hand position A2 precedes B1.

Comparing “Reference Angle α” values of the instrument positions under test with an overall mean (all instrument and hand positions aggregated, black dashed line in Figures 7, 8) shows three instrument positions A1, A2, and B1 above average and two instrument positions Free and B2 below average for sixth hand position. For second position, one instrument position (A1) is above average, position A2 is at the same level as the overall mean, and the remaining instrument positions B1, Free, and B2 are below average. Overall mean values are approx. 4.3 points below instrument position A1. This applies both to the entire duration of the 16-s tune (see Table 4 below) and the moments when the fourth finger normal and fourth finger low are played (see Table 6 below).

**Table 4:** Descriptive statistics for Reference Angle α (elbow compensation movement for the entire 16-s tune.

Instrument position	*N*	Mean	Min.	Max.	*SD*
By instrument position and for sixth hand position (Figure 7)					
A1	60	25.64	22.0	32.4	2.476
A2	60	22.86	19.1	29.4	2.110
B1	60	23.39	18.7	32.0	2.729
B2	60	19.78	15.4	28.7	2.646
Free	60	20.68	15.1	28.4	2.831
**Overall mean for all instrument and hand positions (dashed line --- in Figures 7, 8**)					
Overall mean	450	21.38	12.0	32.0	3.779
**By instrument position and second hand position (Figure 8)**					
A1	30	23.25	19.7	29.6	2.325
A2	30	21.35	16.7	27.4	2.356
B1	30	18.68	13.4	26.2	2.729
B2	30	16.27	11.7	22.7	2.688
Free	30	16.48	11.6	23.4	2.946

Multiple regression analysis for “Reference Angle α” for sixth hand position shows highly significant differences between violin position A1 and all other instrument positions (*N* = 60, *p* < 0.001, r^2^ = 0.398), thereby explaining nearly 40% of elbow/upper arm compensation movement variance when considering all measurements in sixth hand position for a given instrument position. Results for second hand position follow the same pattern (*N* = 30, *p* < 0.001, r^2^ = 0.529), however, with significance levels for instrument A2 being *p* < 0.01. R^2^ for this model is 0.592, thereby explaining 59% of elbow/upper arm compensation movement variance when considering all measurements in second position for a given instrument position. For detailed regression analysis results please refer to Supplementary Table S8.

For sixth hand position, a *post hoc* multiple comparison of means (Scheffé) shows that, for all hand positions united (*N* = 300), mean values between single instrument positions for the entire 16-s tune differ highly significantly between each other (*p* < 0.001) except between instrument positions A2 and B1 as well as B2 and Free (non-significant).

For second hand position, a *post hoc* multiple comparison of means (Scheffé) shows that, for all hand positions united (*N* = 300), mean values between single instrument positions for the entire 16-s tune differ highly significantly between each other (*p* < 0.001) except between instrument positions A2 and B1 (*p* < 0.01), B2 and B1 (*p* < 0.05) as well as Free and B1 (*p* < 0.05). Differences between instrument positions A1 and A2 as well as B2 and Free are non-significant.

#### 4.2.2. Influence of sixth and second hand position on shoulder compensation movement by instrument position (“Coord x”/“Coord y”)

For shoulder motion data (all points in time aggregated, see section 3.4.2) at the level of sixth and second hand position, highest values are observed for instrument position A1 and lowest for B2 with a distinctive gradation of values, i.e., A1 > A2 > B1 > Free>B2. This gradation applies both to shoulder protraction (“Coord x”) as well as to shoulder elevation (“Coord y”). Values for protraction are higher than for elevation (see Table 5 below).

**Table 5:** Descriptive statistics for shoulder compensation movements by hand and instrument position and compared with the overall mean of values for protraction (“Coord x”) and elevation (“Coord y”).

Instrument position	*N*	Mean	Min.	Max.	*SD*
Sixth position, shoulder protraction (“Coord x”)					
A1	300	21.8	6.4	37.8	7.539
A2	300	14.0	4.0	25.8	5.750
B1	300	8.6	1.0	18.0	4.512
B2	300	4.7	−0.3	14.6	3.521
Free	300	9.1	−3.1	25.3	6.736
**Sixth position, shoulder elevation (“Coord y”)**					
A1	300	5.8	0.0	13.3	2.998
A2	300	4.7	0.5	10.8	2.608
B1	300	2.4	−1.2	6.8	2.156
B2	300	2.3	−0.8	9.7	2.048
Free	300	5.7	−1.0	17.5	4.568
**Overall mean for all instrument and hand positions**					
Shoulder protraction overall (“Coord x”)	450	11.9	−3.7	37.3	8.331
Shoulder elevation overall (“Coord y”)	450	3.9	−1.8	15.3	3.219
**Second position, shoulder protraction (“Coord x”)**					
A1	150	13.0	3.5	29.8	6.262
A2	150	8.1	−1.0	18.7	4.276
B1	150	3.9	−2.5	10.6	3.060
B2	150	1.8	−5.0	7.2	2.795
Free	150	7.7	−4.4	22.0	6.272
**Second position, shoulder elevation (“Coord y”)**					
A1	150	3.5	0.6	17.2	3.178
A2	150	2.5	−0.2	6.2	1.757
B1	150	1.7	−3.2	6.1	2.031
B2	150	1.8	−1.5	7.2	2.367
Free	150	5.1	−2.0	13.8	3.531

For sixth hand position, multiple regression analysis for shoulder protraction (“Coord x”) shows highly significant differences between violin position A1 and all other instrument positions (*N* = 60, *p* < 0.001, r^2^ = 0.711), thereby explaining 70% of shoulder protraction variance when considering all measurements in sixth hand position for a given instrument position. For shoulder elevation (“Coord y”), differences between violin positions A1 and B1 as well as B2 are highly significant (*N* = 60, *p* < 0.001, r^2^ = 0.456), and the difference between A1 and instrument positions A2 and Free are non-significant. R^2^ for this model explains 45% of shoulder elevation variance.

For second hand position, multiple regression analysis for shoulder protraction (“Coord x”) shows highly significant differences between violin position A1 and all other instrument positions (*N* = 30, *p* < 0.001, r^2^ = 0.400), explaining 40% of shoulder protraction variance. For shoulder elevation (“Coord y”), significance levels are reached for instrument position B1 (*N* = 30, *p* < 0.01, r^2^ = 0.189) as well as B2 and Free (*N* = 30, *p* < 0.05, r^2^ = 0.189). The difference between instrument position A1 and A2 is non-significant (see Supplementary Table S9).

For sixth hand position, a *post hoc* multiple comparison of means (Scheffé) shows that for shoulder protraction (“Coord x”), mean values between single instrument positions differ highly significantly between each other (*p* < 001) and significantly between instrument positions B1 and B2 (*p* < 0.05) as well as B2 and Free (*p* < 0.010). The difference between instrument position B1 and Free is non-significant.

For shoulder elevation (“Coord y”), highly significant differences between single instrument positions are observed for A1 and B1 as well as B2, B1, and A1, B1 and A1 as well as Free and B2 and A1 and Free (*p* < 0.001). The differences between A2 and B1 as well as B2 and A2 B2 reach the next-highest significance levels (*p* < 0.010). The remaining comparisons are non-significant. For further reference, please refer to Supplementary Table S10.

For second hand position, the same comparison of means for shoulder protraction (“Coord x”) shows highly significant differences for A1 and B1 as well as B2, A2 and B2 and B2 and Free (*p* < 0.001). Comparisons between A1 and A2 as well as Free, A2 and B1 as well as A1 and Free reach the next-highest significance levels (*p* < 0.010). The remaining comparisons are non-significant.

For shoulder elevation (“Coord y”), highly significant differences are observed in two cases: B1 and Free and B2 and Free (*p* < 0.001). The difference between A2 and Free reaches the next-highest significance levels (*p* < 0.010). The remaining comparisons are non-significant. For further reference, please refer to Supplementary Table S10.

### 4.3. Results for specific use of the fourth fingers

#### 4.3.1. Influence of the fourth fingers on elbow/upper arm compensation movements (“Reference Angle ɑ”) by hand and instrument position

For the specific time segments pertaining to fourth finger normal and fourth finger high, results follow the same pattern as for the over 16 s-tune (see section 4.1), in that the highest values for “Reference Angle ɑ” are reported for violin position A1 compared to all other instrument positions and for both hand positions and the gradation between the instrument positions is identical, though at higher levels (see Table 6 below):

**Table 6:** Descriptive statistics for Reference Angle α (elbow/upper arm) compensation movement for the specific time segments when the fourth fingers are played.

Fourth fingers for Instrument and sixth hand position (Figure 7)					
Fourth finger normal (time segments “a,” sec. 2.000–3.995 and 10.000–11.995)	*N*	Mean	Min.	Max.	*SD*
A1	60	26.26	22.7	33.0	2.421
A2	60	23.34	19.6	29.7	2.087
B1	60	24.23	19.6	33.2	2.793
B2	60	20.44	16.0	29.8	2.722
Free	60	21.36	15.6	29.0	2.876
Fourth finger high (time segment “b,” sec. 14.000–15.995)					
A1	60	26.62	22.8	33.7	2.509
A2	60	23.54	19.6	29.7	2.033
B1	60	24.61	19.8	34.0	2.816
B2	60	20.81	16.3	29.6	2.591
Free	60	21.78	16.1	29.2	2.805
**Overall mean for all instrument and hand positions (dashed line --- in Figures 7, 8)**					
Fourth finger normal (time segments “a”)	450	21.91	12.0	33.0	3.865
Fourth finger high (time segment “b”)	450	22.28	12.0	34.0	3.806
**Fourth fingers for instrument and second hand position (Figure 8)**					
Fourth finger normal (time segments “a”)					
A1	30	23.46	19.9	29.6	2.337
A2	30	21.55	16.9	27.8	2.368
B1	30	19.01	13.4	26.7	2.798
B2	30	16.58	11.6	23.5	2.839
Free	30	16.86	11.9	23.6	3.005
Fourth finger high (time segment “b”)					
A1	30	23.72	20.2	31.0	2.427
A2	30	21.68	18.0	27.8	2.285
B1	30	19.64	14.6	29.9	3.060
B2	30	17.08	12.5	23.0	2.568
Free	30	17.40	12.0	23.6	3.059

Multiple regression analysis for “Reference Angle α” in the specific moments when the fourth fingers play shows highly significant differences between violin position A1 and all other instrument positions for time segments for fourth finger normal (*N* = 90, *p* < 0.001, r^2^ = 0.329) and fourth finger high (*N* = 90, *p* < 0.001, r^2^ = 0.324), thereby explaining 33 and 32% of elbow/upper arm compensation movement variance when considering all hand positions for a given instrument position.

Results for sixth hand position shows highly significant differences between A1 and all other instrument positions, both for fourth finger normal (*N* = 60, *p* < 0.001, r^2^ = 0.393) and fourth finger high (*N* = 60, *p* < 0.001, r^2^ = 0.395), thereby explaining between 39 and 40% of elbow/upper arm compensation movement variance when considering all measurements in sixth position for a given instrument position.

Results for second hand position are comparable to the abovementioned categories for time segments for fourth finger normal (*N* = 30, *p* < 0.001, r^2^ = 0.506) and fourth finger high (*N* = 30, *p* < 0.001, r^2^ = 0.476) except for A2 (*p* < 0.01), R^2^ thereby explaining between 47 and 50% of elbow/upper arm compensation movement variance when considering all measurements in second position for a given instrument position (see Supplementary Table S11).

A *post hoc* multiple comparison of means (Scheffé) shows that, for the specific time segments of the normal and high fourth finger for all hand positions united (*N* = 450), mean values between single instrument positions for the entire tune differ highly significantly among each other (*p* < 0.001) except for A2 to B1 and B2 to Free (non-significant). The same pattern applies to the sequences played in sixth hand position and the time segments where the normal and the high fourth finger are played. For further reference, please refer to Supplementary Table S12.

For the normal fourth finger played in second hand position, the comparison of means shows the following pattern of differences between instrument positions:

Within the “A-type” instrument positions, A1 differs highly significantly from all other positions (*p* < 0.001) except for A2 (non-significant). Position A2 differs highly significantly from positions B2 and Free (*p* < 0.001), significantly from B1 (*p* < 0.05), and not from A1 (non-significant).

Within the “B-type” instrument positions, B1 differs highly significantly from A1 (*p* < 0.001) and significantly from A2 and B2 (*p* < 0.05) but not from Free (non-significant). B2 differs highly significantly from positions A1 and A2 (*p* < 0.001), significantly from B1 (*p* < 0.05), and not from position Free (non-significant). Instrument position Free differs highly significantly from positions A1 and A2 (*p* < 0.001) and not from B1 (*p* < 0.05) and B2 (non-significant). For further reference, please refer to Supplementary Table S12.

For the high fourth finger played in second hand position, the comparison of means shows the following pattern of differences between instrument positions:

Within the “A-type” instrument positions, A1 differs highly significantly from all other positions (*p* < 0.001) except for A2 (non-significant). Position A2 differs highly significantly from positions B2 and Free (*p* < 0.001), while for all other position differences are non-significant.

Within the “B-type” instrument positions, B1 differs highly significantly from A1 (*p* < 0.001) and significantly from B2 and Free (*p* < 0.05) but not from A2 (non-significant). B2 differs highly significantly from positions A1 and A2 (*p* < 0.001), significantly from B1 (*p* < 0.05), and not from position Free (non-significant). Instrument position Free differs highly significantly from positions A1 and A2 (*p* < 0.001), significantly from B1 (*p* < 0.05), and not from position B2 (non-significant). For further reference, please refer to Supplementary Table S12.

#### 4.3.2. Influence of the fourth fingers on shoulder compensation movements (“Coord x”/”Coord y”) by hand and instrument position

Data for shoulder compensation movements focusing on the points in time when the fourth finger is played show a comparable pattern as reported in section 4.2.2: Values for instrument position A1 are highest and B2 lowest, and the gradation of values between instrument positions is identical. Compared with the overall means for the respective hand positions (grey shaded area in Supplementary Table S13), data suggests that instrument positions with a smaller angle degree for LoAx-CSP and LatAx-HP, as well as the “Free” position are linked to higher values for shoulder protraction and elevation throughout all points in time observed than for instrument position with a larger angle for LoAx-CSP and LatAx-HP.

Further details regarding the influence of the hand position on the degree of shoulder protraction and elevation can be found in the Supplementary Tables S13–S15.

### 4.4. Correlation analyses for target parameters

#### 4.4.1. Correlation between shoulder motion data and elbow/upper arm compensation movement

The linear relationship between “Coord x” (shoulder protraction) and “Reference Angle α”, as well as between “Coord y “(shoulder elevation) and “Reference Angle α” was assessed by computing Pearson’s *r* correlation coefficient for all five instrument positions, both hand positions and all three time points under test.

Instrument position Free yields significant positive correlations between “Coord x” and “Reference Angle α” for all observations. Among the correlations between “Coord y” and “Reference Angle α,” two out of six are significant. Positive correlations between “Coord x” and “Reference Angle α” are also observed for instrument position A2 at the beginning of the tune for sixth hand position (r = 0.304, *p* = 0.018), second hand position (r = 0.372, *p* = 0.043) as well as for second hand position fourth finger normal (r = 0.361, *p* = 0.050) and fourth finger high (r = 0.363, *p* = 0.049). A further significant correlation is reported for instrument position B2 for sixth hand position at the beginning of the tune (r = 0.268, *p* = 0.038).

For further details, please refer to Supplementary Table S16.

#### 4.4.2. Correlation between biomechanical parameters and elbow/upper arm compensation movement

The linear relationship between the biomechanical parameters and “Reference Angle α” was assessed by computing Pearson’s *r* correlation coefficient for all five instrument positions, both hand positions, and all three time points under test.

For “Passive Supination 250 g” and “Passive Supination 500 g”, nine out of 10 statistically significant, negative correlations with “Reference Angle α” are reported for the entire 16-s tune (the correlation for instrument position Free in second hand position is non-significant). This also applies to the normal fourth finger in the case of correlations between “Passive Supination 250 g” and “Reference Angle α”. Correlations between “Passive Supination 500g” and “Reference Angle α” yield eight out of 10 statistically significant, negative correlations (the instrument positions Free and B1 in second hand position are non-significant). For the high fourth finger, seven out of 10 statistically significant, negative correlations with “Reference Angle α” are reported both for “Passive Supination 250 g” and “Passive Supination 500 g” (instrument positions B1, B2 and Free in second hand position are non-significant).

For Passive Thumb Abduction and Reference Angle α, four out of five positive correlations are reported for sixth hand position for the entire 16-s tune and the specific use of the normal and high fourth finger (the instrument position Free is non-significant). An additional positive correlation is reported for second hand position in instrument position A1 for the high fourth finger. For “Finger Length Difference 3-5”, no significant correlations with “Reference Angle α” are reported. For further details, please refer to Supplementary Table S17.

#### 4.4.3. Correlation between biomechanical parameters and shoulder motion data

The linear relationship between “Coord x” (shoulder protraction) and the biomechanical parameters, as well as between “Coord y” (shoulder elevation) and the biomechanical parameters, was assessed by computing Pearson’s *r* correlation coefficient for all five instrument positions, both hand positions and all three time points under test.

A negative correlation between “Coord x” and biomechanical parameter “Passive supination 250 g” is reported for instrument position B2, sixth hand position for the beginning of tune (r = −0.268, *p* = 0.038).

Negative correlations for “Coord y” and biomechanical parameter “Passive supination 250 g” are reported for instrument position A1, sixth position for the beginning of the tune (r = −0.348, *p* = 0.006), instrument position A2, sixth position for the beginning of the tune (r = −0.334, *p* = 0.009), fourth finger normal (r = −0.290, *p* = 0.025) and fourth finger high (r = −0.267, *p* = 0.039), instrument position B1, sixth position for the beginning of the tune (r = −0.380, *p* = 0.003) and second position for the beginning of the tune (r = −0.368, *p* = 0.045) and instrument position B2, sixth position for the beginning of the tune (r = −0.289, p = 0.025), fourth finger normal (r = −0.334, p = 0.009) and fourth finger high (r = −0.290, *p* = 0.024).

## 5. Discussion

### 5.1. Analysis of the data

The spectrum of recommendations on appropriate instrument positioning put forward by teaching traditions over the last four centuries remains very broad and partly contradictory. It could be considered a co-factor contributing to the high incidence of musculoskeletal complaints to this day. Therefore, this study investigated the effects of position changes in a violin’s sideward orientation (LoAx-CSP) and inclination (LatAx-HP) on the extent of compensatory movements of the left upper extremity: Using pre-defined violin positions while playing a standardized 16-s tune, the hypothesis was confirmed that a reduction of LoAx-CSP and LatAx-HP by 30° (i.e., within a common inter-/intraindividual range of variability, including the individuals’ habitual, “free” position without the support of the experimental set-up) significantly and independently increased the magnitude of compensatory movement of the left elbow and upper arm (“Reference Angle α”) and left shoulder (protraction, “Coord x”/elevation, “Coord y”).

As reported for the parameters in this publication, the same observation has already been made at the objective level of muscle activation and the subjective level of perceived effort. Those published results confirm comparable patterns of changes of muscle activation and subjectively perceived muscular effort in the five instrument positions under test (Hildebrandt et al., 2021). This applies particularly to the pectoralis major muscle, which is significantly involved in the compensatory movement of the left upper arm and elbow in front of the trunk. Changing LatAx-HP from 50° to 20° (i.e., changing from position “2” to “1”) and LoAx-CSP from 50° to 20° (i.e., changing from position “B” to “A,” see Table 1 above) not only increased the level of muscle activation and subjectively felt effort; it also increased the extent of the compensatory movements of both the elbow/upper arm and the shoulder. These results are confirmed at all three levels investigated in the data presented in this paper: The instrument positioning (i.e., positions A1 through Free), hand positioning (i.e., sixth and second hand position), and finger positioning with a focal point lying on the fourth fingers as a special point of interest and relevance for violin performance.

In longer and more complex real-life endurance challenges (including co-factors such as the chin-and shoulder rests), the patterns and degrees of compensatory movements and the resulting effort levels are likely to be either more pronounced or variegated than would be the case in the short tunes tested in a laboratory environment. Results for the correlation between shoulder motion data and elbow/upper arm compensation movement (see section 4.4.1) could indicate that players find ways how to adapt their playing position. This adaptive process appears to lead to different combinations of compensation patterns and may benefit them when playing in a normal environment. This aspect, however, requires further investigation.

Previous studies have pointed to the specific role of the muscles associated to the shoulder girdle triggering compensatory movements (Steinmetz et al., 2008; Steinmetz, 2016; Möller et al., 2018). It appears that the more proximal arm muscles play a critical role in muscular workload in violin playing [i.e., through limited isometric endurance during longer tasks with likely critically reduced blood and O2-supply (Hollmann and Strüder, 2009)], while more distal tasks appear to be more dynamic and involve only shorter isometric components. While previous research focuses predominantly on muscle activation levels and variability while violinists perform tasks on their own instrument (see Background section), results linked to this body of research (Hildebrandt et al., 2021) offer insight into muscle activation levels in relation to positional effects generated by standardized instrument positions. In this study, the selection of muscles and respective electrode placement mirrors other focal points in research, such as the role of the pectoralis major muscle in violin performance. By means of the 2D video motion analysis, data indirectly linked to functional aspects of the trapezius as well as the levator scapulae muscle, which are often in focus in case of task-specific health issues (Chan et al., 2000; Spahn et al., 2001; Berque and Gray, 2002; Hildebrandt and Nübling, 2004; Wagner, 2005; Chi et al., 2020; Gembris et al., 2020; Mann et al., 2021), were obtained. In line with this paper’s finding on movement patterns of the left upper extremity, results for muscle activation levels suggest that, in addition to muscles focused on in other studies, the pectoralis major muscle should be equally considered when either identifying possible causes for task-specific health issues or when advising performers in view of the prevention thereof. Thus, this study’s results re-confirm and extend our knowledge regarding high-stress risks in the shoulder girdle, such as playing-related health disorders, including musculo-fascial overload and shoulder impingement syndromes. The number of studies dedicated to electromyographic (EMG) measurements of violinists is growing and examines a broad spectrum of relevant aspects, such as the influence of ergonomics, anthropometrics and repertoire (Philipson et al., 1990; Cattarello et al., 2017, 2018; Kok et al., 2019; Chi et al., 2020; Mann et al., 2021), the comparison between muscle activation levels in healthy violinists and those reporting task-specific health problems (Spahn et al., 2001; Berque and Gray, 2002; Hildebrandt and Nübling, 2004), muscular variability, endurance and fatigue aspects of violin performance (Wagner, 2005; Gembris et al., 2020). In contrast, research on the comparison between subjectively perceived effort levels and objective data on muscle activation when playing the violin appears to be scarcer (Chan et al., 2000; Hildebrandt et al., 2021).

This study’s sub-hypothesis (“the lower the passive supination ability and passive thumb spreading and the shorter the length of the little finger in comparison relative to the middle finger, the more compensation movements of the upper extremity”) was confirmed for passive supination, not confirmed for passive thumb spreading and not confirmed the length of the little finger in comparison relative to the middle finger. The results on the influence of these biomechanical factors on compensation movements show that supination ability correlates negatively with the extent of elbow/upper arm compensatory movements in all instrument positions, hand positions, and positions of the fourth fingers. Especially the fourth finger on the violin’s lowest string, this indicates the relevance of passive supination for the fingers’ reach: a reduced degree of supination ability seems to provoke increased elbow or upper arm movements in front of the trunk, thereby affecting proximal structures such as the shoulder girdle and its musculature.

The ability to spread the thumb also appears to be relevant for the fingers’ reach when playing the instrument. In contrast to our sub-hypothesis, it seems to allow a higher degree of compensation movements of elbow/upper arm, and shoulder but does not reach a significant level when correlated with the player’s usual instrument position (“Free”). However, significance levels of the compensation movements in the standardized test positions A1 through B2 increase with specific playing requirements during the tune (i.e., the use of the fourth finger in higher hand positions). The ability to spread the thumb cannot be clearly captured as enabling or disabling the fingers range based on data collected in this study. Also, the contradictory fact that correlations between passive thumb abduction and elbow/upper arm compensation are positive while the same biomechanical parameter correlated with data for shoulder elevation would lead to isolated cases of negative correlation calls for further investigations.

A surprising fact is that a small fifth finger (i.e., the fourth finger in violin playing), which is set back more clearly relative to the third finger, does not show any significant correlation with compensatory movements of elbow/arm and shoulder. One explanation for this could be that the differences measured in millimeters for the fifth finger are too small to trigger/elicit significantly different levels of compensation movements, which would occur in the finger stretching out along its longitudinal axis when approaching the string diagonally. Another explanation could be that players with relatively short fifth fingers are already accustomed to applying a combination of compensation movement patterns relevant for successfully performing in a given instrument or hand position before starting to play. A sub-analysis of existing data or future studies will become necessary to further clarify this aspect.

Taken together, the present results suggest that differences in effort between typical violin positions can be captured not only by objective EMG measurements and subjective BORG assessments (Hildebrandt et al., 2021), but also in the extent of compensatory movements of the elbow, upper arm and shoulder. Based on the results, a recommendation could be issued that violin position A1, in which both LoAX-CSP and LatAX-HP angles are decreased, seems significantly disadvantageous in terms of the overall effort required, and should be assumed for a limited time only. This aspect becomes particularly relevant when focusing on historically informed performance practices, where instrument positions resembling A1 are assumed (Rônez-Kubitschek, 2012). As such, results from this body of research may contribute to the choice of individually beneficial instrument positioning based on objective findings and creating choices along a spectrum of options. Results also add to the growing body of research aiming to understand the interdependency between violin performance and playing technique, relevant muscles involved, and contributing factors for developing task-specific health problems in this musician population.

An interesting detail is that, in contrast to the results for EMG and Borg measurements in the same five instrument positions (Hildebrandt et al., 2021), the extended fourth finger provokes only minimally more compensatory movements of the elbow/upper arm than the normal fourth finger. Furthermore, the more inclined instrument position (A2) seems to restrict the compensatory protraction movement of the shoulder, but at the same time, provokes more compensatory movements of the elbow and arm.

As expected, more compensatory movements occur synchronously with specific technical requirements of playing (e.g., performing in a high hand position or using the fourth finger). On one hand, this fact could be considered as an indication for a targeted and dynamic repositioning of the instrument in view of adding to the ease of performing pieces with an increased use of high registers and fourth finger, which is often the case in highly virtuosic and therefore challenging compositions. On the other hand, these findings may contribute to the individualized choice and adaptation of ergonomic aids and concepts of rest during training to prevent task-specific health issues.

### 5.2. Limitations of the study

The experimental setting for the assessment of violin positions A1-B2 excluded the supporting of the instrument by means of the active use of the arm, head, or shoulder, but did include the effort required to raise the arm. The latter’s effect on arm and shoulder compensatory movements was not quantified in terms of its contribution to compensatory movements varying between A1-B2. Better separation between violin support and actual playing is a challenge for future studies. However, a significant proportion of the variance in arm and shoulder compensation movements was due to the positions under test. It can be expected that during prolonged static tasks as observable in a real-life environment, the overall effects of LoAx-CSP and LatAx-HP on the target parameters examined are likely to become more pronounced, and that smaller ergonomic disadvantages may also become more apparent in the degree of compensation movements. In this respect, future studies should not only record longer phases of playing (as they are common in orchestral playing, for example) but should also take a higher-resolution approach by examining the effects of smaller angular deviations from A1 (10 or 20 instead of 30°) in comparative tests.

A general limitation of the present study is that the participants played on the two lower strings, with neither vibrato, hand position shifts or changes in the position of the thumb, elbow, and head. These factors might contribute to subtle postural adjustments while playing and add to the improvement of comfort and possibly skill. In the actual practice of violin teaching and playing, the individual violin position is chosen as an individually preferred combination of the instrument’s inclination and sideward orientation, as mirrored in the standardized violin positions investigated in this study. Also, a performer’s learning biography and performance preferences should be viewed as a dynamic transition along the spectrum covered by these standardized positions. Further variations within these standardized violin positions had to be excluded in the context of this study to provide a solid scientific basis.

Possibly, more importantly, the exclusion of the use of the bow and its weight when running the tests may have influenced the position and comfort of the left arm in a currently undefined way: The length of the right arm, the point of contact with the strings, and its influence on the left arm and hand adjustments to dynamics, sound quality, bow speed, etc. were not required. Therefore, a note should be added to the data presented that further testing should be devoted to more complex conditions involving bowing tasks and switching between additional strings, hand or finger positions, and consideration of anthropometric characteristics (Ackermann and Adams, 2003; Wagner, 2005; Wagner, 2012). Future studies will also aim to expand the technological possibilities of generating shoulder motion data in analogy to the 3D motion capture system used to collect data for “Reference Anlge α” to exclude potential inter-rater variability common for analogue evaluation methods and as applied to shoulder motion data collection for “Coord x” and “Coord y”.

### 5.3. Translational aspects

Throughout the centuries-long tradition of violin playing and teaching, a wide range of recommendations and traditions regarding instrument positioning have been propagated by violin pedagogues (Flesch, 1978; Galamian, 1983; Rostal, 1993; Rônez-Kubitschek, 2012) and first scientific approaches (Szende and Nemessury, 1971). Each recommendation issued by a respective school (e.g., German, Italian, and French; Rônez-Kubitschek, 2012) influences the sidewards orientation of the instrument (LoAx-CSP) and the inclination of the instrument (LatAx-HP). Despite the limitations mentioned above, the present study provides further insights into the possible effects of violin positioning on parameters relevant to musicians’ health. The final choice of instrument positioning remains a decision that the violinist makes with a teacher or therapist. However, when a violin position is needed that involves a lower degree of shoulder and arm compensatory movement or muscle activation, data can provide initial steps towards more targeted decision-making. This could, for example, prove useful when recovering from a task-specific injury or in case of limited supination ability.

In addition, the data may help to maintain (proximal) muscle activation at a lower level and thus extend the duration of the performance before the onset of muscle effort at higher levels. This aspect can be important in performance contexts such as when playing in an orchestra: The violin sometimes needs to be held and supported for several hours, with trade-offs between sitting position, the ergonomic challenges of making music together with a stand partner and ensuring contact with, section leaders and the conductor. For players in the context of historically informed performance practice, the choice of positioning may be adapted to the requirements of a particular musical structure, e.g., high registers and the number of hand position changes.

It may also be that the instrument does not necessarily need to be played in the objectively easiest position, given a violinist’s expressive quality or a subjectively perceived optimum. Some violinists may choose to play in a position that involves slightly greater compensatory movement and muscle activation if it benefits the level of expression or provides a temporary sense of greater stability to master a passage in performance. However, health issues may need to be addressed depending on how long this condition lasts.

As all participants were young, healthy students who had no musculoskeletal complaints at the time of measurement, the results may be insufficiently representative of a population with a high prevalence of musculoskeletal complaints due to compensatory movements, such as for example orchestral players (Berque and Gray, 2002; Steinmetz et al., 2008; Vinci et al., 2015; Kochem and Silva, 2017). A more detailed picture of the causative factors for these complaints could also include other influences, such as individual patterns of posture and movement and practice habits, including approaches to taking breaks during practicing and working. Similarly, despite initial encouraging results and observations based on this study (Hollmann and Strüder, 2009; Hildebrandt et al., 2021; Newly Developed Multi Adaptable Chin Rest, Model Zuerich, 2017), further research should be conducted to assess the transferability of this study’s findings to classroom, counseling, and therapy settings in terms of their validity. Gender effects and an even broader focus on anthropometric co-factors (Ackermann and Adams, 2003; Wagner, 2005; Wagner, 2012) should also be considered in future studies.

## 6. Conclusion

The central finding of this study is that when the sideward orientation of the instrument (LoAx-CSP angle) and the inclination of the instrument (LatAx-HP angle) are decreased by 30°, the compensatory movements of the violinist’s left elbow/upper arm and shoulder increase highly significantly and independently of each other and are additionally increased by biomechanical factors such as reduced supination ability and increased thumb spreading ability. Furthermore, there is an increase in compensatory movements in synchrony with playing technique requirements such as higher positions and the use of the little finger. Since the present tests were relatively short (16 s. each), more significant effects are likely to be expected in the case of longer-lasting professional tasks, even with smaller changes in angle. Although these findings are preliminary, they can serve as indications for favorable instrument positions and training concepts. It should be kept in mind that compensatory movements result from a highly complex interplay between individual elements of violin posture, the various temporally coordinated movement demands of the left and right hands and general expressive intentions.

## Data availability statement

The datasets presented in this article are not readily available because parts of data sets currently in evaluation process for further publications. Requests to access the datasets should be directed to oliver.margulies@zhdk.ch.

## Ethics statement

The studies involving human participants were reviewed and approved by Canton of Zürich Ethics Committee (Project number: KEK-ZH-Nr. 2014-0008). The patients/participants provided their written informed consent to participate in this study.

## Author contributions

HH: grant applicant and project leader, overall management, and specialist supervision of all project phases. OM: project co-leader, organization, practical and specialist supervision of all project phases, and preparation of data for analysis. MN and WV: methodology, data collection, data analysis, and data interpretation. WH: consultation on functional anatomical biomechanical matters, data analysis and interpretation, and specialist supervision. All authors contributed to the article and approved the submitted version.

## Funding

This research was carried out thanks to a grant from the Swiss National Science Foundation (grant number: 105316_153210) and financial support from the Ernst Göhner Stiftung (project number: 2013-2635).

## Conflict of interest

The authors declare that the research was conducted in the absence of any commercial or financial relationships that could be construed as a potential conflict of interest.

## Publisher’s note

All claims expressed in this article are solely those of the authors and do not necessarily represent those of their affiliated organizations, or those of the publisher, the editors and the reviewers. Any product that may be evaluated in this article, or claim that may be made by its manufacturer, is not guaranteed or endorsed by the publisher.
