# Supplementary material for: Determining factors for compensatory movements of the left arm and shoulder in violin playing
Source: Front Psychol. 2023 Jan 23;13:1017039. doi: 10.3389/fpsyg.2022.1017039 (PMC9901209; doi:10.3389/fpsyg.2022.1017039)
Supplement: Supplementary file 1 [file Data_Sheet_1.pdf]

Supplementary Material for Chapter 4.1.1

Table S1

Time Segment	Instrument Position	Mean	N	Min.	Max.	SD
**Entire tune**	**A1**	24.84	90	20	32	2.666
	**A2**	22.36	90	17	29	2.296
	**B1**	21.82	90	13	32	3.515
	**B2**	18.61	90	12	29	3.124
	**Free**	19.28	90	12	28	3.479
	**Overall (- - -)**	21.38	450	12	32	3.779

Table A: Degree of elbow/upper arm compensation, expressed as “Reference Angle α”

Table S2

		Non-standardized Coefficients		Standardized Coefficients		
**Time Segment**	**Violin Position**	**Regression Coefficient B**	**Standard Error**	**Beta**	**Sig.**	**R^2 Model**
**All Hand Positions (N=90 per instrument position)**						
**Entire tune**	**A1**	24.840	0.322			0.353
	**A2**	-2.481	0.455	-0.263	p<0.001	
	**B1**	-3.019	0.455	-0.320	p<0.001	
	**B2**	-6.226	0.455	-0.660	p<0.001	
	**Free**	-5.558	0.455	-0.589	p<0.001	

Table B: Regression Analysis for “Reference Angle α” by instrument Position

Table S3

Time Segment			Mean Difference	Standard Error	Sig.	95% CI Lower Bound	95% CI Upper Bound
Entire tune	A1	A2	2,481*	0.455	p<0.001	1.07	3.89
		B1	3,019*	0.455	p<0.001	1.61	4.43
		B2	6,226*	0.455	p<0.001	4.82	7.63
		Free	5,558*	0.455	p<0.001	4.15	6.97
	A2	A1	-2,481*	0.455	p<0.001	-3.89	-1.07
		B1	0.538	0.455	0.844	-0.87	1.95
		B2	3,745*	0.455	p<0.001	2.34	5.15
		Free	3,077*	0.455	p<0.001	1.67	4.48
	B1	A1	-3,019*	0.455	p<0.001	-4.43	-1.61
		A2	-0.538	0.455	0.844	-1.95	0.87
		B2	3,207*	0.455	p<0.001	1.80	4.61
		Free	2,539*	0.455	p<0.001	1.13	3.95
	B2	A1	-6,226*	0.455	p<0.001	-7.63	-4.82
		A2	-3,745*	0.455	p<0.001	-5.15	-2.34
		B1	-3,207*	0.455	p<0.001	-4.61	-1.80
		Free	-0.668	0.455	0.707	-2.08	0.74
	Free	A1	-5,558*	0.455	p<0.001	-6.97	-4.15
		A2	-3,077*	0.455	p<0.001	-4.48	-1.67
		B1	-2,539*	0.455	p<0.001	-3.95	-1.13
		B2	0.668	0.455	0.707	-0.74	2.08

Table C: Post-hoc, multiple comparison of means (Scheffé) for "Reference Angle α" by instrument Position

Supplementary Material for Chapter 4.1.2

Table S4

Shoulder Protraction ("Coord x")					
**Instrument Position**	**N**	**Mean**	**Minimum**	**Maximum**	**SD**
**A1**	90	22.54	8.2	37.3	7.417
**A2**	90	14.08	4.0	25.3	5.471
**B1**	90	8.91	0.5	18.2	4.503
**B2**	90	4.75	-0.7	15.5	3.488
**Free**	90	9.07	-3.7	23.3	6.723
**All Instrument Positions Aggregated**					
**"Coord x" Overall**	450	11.87	-3.7	37.3	8.339
**"Coord y" Overall**	450	3.86	-1.8	15.3	3.222
**Shoulder Elevation ("Coord y")**					
**A1**	90	5.66	-0.2	15.3	3.287
**A2**	90	4.24	0.7	9.3	2.315
**B1**	90	2.22	-1.8	6.8	2.233
**B2**	90	1.94	-1.2	5.8	1.901
**Free**	90	5.27	-1.7	15.0	3.971

Table D: Descriptive statistics for shoulder compensation movements by direction and instrument position

Table S5

Violin Position	Non-standardized Coefficients: Regression Coefficient B	Non-standardized Coefficients: Standard Error	Standardized Coefficients: Beta	Sig.	R^2 Model
"Coord x" (Shoulder Protraction), all Hand Positions united (N=449)					
**A1**	22.544	0.601			0.536
**A2**	-8.467	0.850	-0.407	p<0.001	
**B1**	-13.633	0.850	-0.655	p<0.001	
**B2**	-17.794	0.850	-0.855	p<0.001	
**Free**	-13.472	0.850	-0.647	p<0.001	
"Coord y" (Shoulder Elevation), all Hand Positions united (N=449)					
**A1**	5.656	0.300			0.226
**A2**	-1.417	0.424	-0.176	p<0.01	
**B1**	-3.439	0.424	-0.427	p<0.001	
**B2**	-3.711	0.424	-0.461	p<0.001	
**Free**	-0.389	0.424	-0.048	n.s.	

Table E: Regression Analysis for "Coord x" (shoulder protraction) and "Coord y" (shoulder elevation) by instrument position

Table S6

Multiple Comparison of Means (Scheffé) for Shoulder Protraction ("Coord x")						
					95% CI	
Time Segment		Mean Difference	Standard Error	Sig.	Lower Bound	Upper Bound
**A1**	**A2**	8,467*	0.850	p<0.001	5.84	11.10
	**B1**	13,633*	0.850	p<0.001	11.00	16.26
	**B2**	17,794*	0.850	p<0.001	15.16	20.42
	**Free**	13,472*	0.850	p<0.001	10.84	16.10
**A2**	**A1**	-8,467*	0.850	p<0.001	-11.10	-5.84
	**B1**	5,167*	0.850	p<0.001	2.54	7.80
	**B2**	9,328*	0.850	p<0.001	6.70	11.96
	**Free**	5,006*	0.850	p<0.001	2.38	7.64
**B1**	**A1**	-13,633*	0.850	p<0.001	-16.26	-11.00
	**A2**	-5,167*	0.850	p<0.001	-7.80	-2.54
	**B2**	4,161*	0.850	p<0.001	1.53	6.79
	**Free**	-0.161	0.850	1.000	-2.79	2.47
**B2**	**A1**	-17,794*	0.850	p<0.001	-20.42	-15.16
	**A2**	-9,328*	0.850	p<0.001	-11.96	-6.70
	**B1**	-4,161*	0.850	p<0.001	-6.79	-1.53
	**Free**	-4,322*	0.850	p<0.001	-6.95	-1.69
**Free**	**A1**	-13,472*	0.850	p<0.001	-16.10	-10.84
	**A2**	-5,006*	0.850	p<0.001	-7.64	-2.38
	**B1**	0.161	0.850	1.000	-2.47	2.79
	**B2**	4,322*	0.850	p<0.001	1.69	6.95
**Multiple Comparison of Means (Scheffé) for Shoulder Elevation ("Coord y")**						
					95% CI	
Time Segment		Mean Difference	Standard Error	Sig.	Lower Bound	Upper Bound
**A1**	**A2**	1,417*	0.424	p<0.05	0.10	2.73
	**B1**	3,439*	0.424	p<0.001	2.13	4.75
	**B2**	3,711*	0.424	p<0.001	2.40	5.02
	**Free**	0.389	0.424	0.933	-0.92	1.70
**A2**	**A1**	-1,417*	0.424	p<0.05	-2.73	-0.10
	**B1**	2,022*	0.424	p<0.001	0.71	3.34
	**B2**	2,294*	0.424	p<0.001	0.98	3.61
	**Free**	-1.028	0.424	0.211	-2.34	0.29
**B1**	**A1**	-3,439*	0.424	p<0.001	-4.75	-2.13
	**A2**	-2,022*	0.424	p<0.001	-3.34	-0.71
	**B2**	0.272	0.424	0.981	-1.04	1.59
	**Free**	-3,050*	0.424	p<0.001	-4.36	-1.74
**B2**	**A1**	-3,711*	0.424	p<0.001	-5.02	-2.40
	**A2**	-2,294*	0.424	p<0.001	-3.61	-0.98
	**B1**	-0.272	0.424	0.981	-1.59	1.04
	**Free**	-3,322*	0.424	p<0.001	-4.64	-2.01
**Free**	**A1**	-0.389	0.424	0.933	-1.70	0.92
	**A2**	1.028	0.424	0.211	-0.29	2.34
	**B1**	3,050*	0.424	p<0.001	1.74	4.36
	**B2**	3,322*	0.424	p<0.001	2.01	4.64

Table F: Post-hoc, multiple comparison of means (Scheffé) for shoulder protraction ("Coord x") and shoulder elevation ("Coord y") by instrument position (overall mean for all time points recorded)

Supplementary Material for Chapter 4.1.3

Table S7

**Instrument Position**	**Biomechanical Parameter**	**“Reference Angle α”**		**Shoulder Protraction (“Coord x”)**		**Shoulder Elevation (“Coord y”)**	
		r	***p***	***r***	***p***	***r***	***p***
**A1** (N=90)	Finger Length Diff 3_5	0.008	0.940	-0.216	<.05 (*)	0.074	0.487
	Passive Supination 250g	-0.469	<.001 (***)	0.029	0.783	-0.320	<.05 (*)
	Passive Supination 500g	-0.490	<.001 (***)	0.040	0.710	-0.268	<.05 (*)
	Passive Thumb Abduction	0.270	<.05 (*)	0.171	0.108	-0.107	0.316
**A2** (N=90)	Finger Length Diff 3_5	0.015	0.885	-0.079	0.462	0.185	0.081
	Passive Supination 250g	-0.525	<.001 (***)	0.020	0.850	-0.287	<.01 (**)
	Passive Supination 500g	-0.529	<.001 (***)	-0.060	0.573	-0.206	0.051
	Passive Thumb Abduction	0.255	<.05 (*)	0.126	0.236	-0.102	0.340
**B1** (N=90)	Finger Length Diff 3_5	-0.002	0.985	-0.158	0.136	0.104	0.328
	Passive Supination 250g	-0.365	<.001 (***)	0.076	0.474	-0.302	<.01 (**)
	Passive Supination 500g	-0.349	<.01 (**)	0.022	0.838	-0.060	0.573
	Passive Thumb Abduction	0.189	0.074	0.051	0.633	-0.217	<.05 (*)
**B2** (N=90)	Finger Length Diff 3_5	0.033	0.759	0.144	0.176	0.244	0.020
	Passive Supination 250g	-0.384	<.001 (***)	0.112	0.294	-0.326	<.01 (**)
	Passive Supination 500g	-0.400	<.001 (***)	0.090	0.398	-0.154	0.147
	Passive Thumb Abduction	0.226	<.05 (*)	0.152	0.154	-0.250	<.05 (*)
**Free** (N=90)	Finger Length Diff 3_5	0.079	0.456	0.121	0.255	0.102	0.340
	Passive Supination 250g	-0.257	<.05 (*)	-0.027	0.803	-0.032	0.765
	Passive Supination 500g	-0.313	<.01 (**)	-0.123	0.249	0.144	0.175
	Passive Thumb Abduction	0.013	0.900	0.223	<.05 (*)	0.077	0.470
**Overall** (N=450)	Finger Length Diff 3_5	0.023	0.633	-0.034	0.472	0.110	<.05 (*)
	Passive Supination 250g	-0.307	<.001 (***)	0.021	0.655	-0.194	<.001 (***)
	Passive Supination 500g	-0.321	<.001 (***)	-0.011	0.821	-0.075	0.112
	Passive Thumb Abduction	0.143	<.01 (**)	0.101	<.05 (*)	-0.077	0.104

Table G: Correlation analysis between biomechanical data and “Reference Angle α” as well as shoulder motion data (“Coord x” and “Coord y”)

Supplementary Material for Chapter 4.2.1

Table S8

Time Segment	Violin Position	N	Non-standardized Coefficients		Standardized Coefficients	Sig.	R^2 Model
			Regression Coefficient B	Standard Error	Beta		
**6th Hand Position (Fig. 9)**							
**Entire tune**	**A1**	60	25.635	0.332			0.398
	**A2**	60	-2.775	0.469	-0.338	p<0.001	
	**B1**	60	-2.244	0.469	-0.273	p<0.001	
	**B2**	60	-5.853	0.469	-0.713	p<0.001	
	**Free**	60	-4.955	0.469	-0.603	p<0.001	
**2nd Hand Position (Fig. 10)**							
**Entire tune**	**A1**	30	23.248	0.478			0.529
	**A2**	30	-1.894	0.676	-0.202	p<0.01	
	**B1**	30	-4.569	0.676	-0.487	p<0.001	
	**B2**	30	-6.973	0.676	-0.743	p<0.001	
	**Free**	30	-6.765	0.676	-0.721	p<0.001	

Table H: Regression analysis for “Reference Angle α” (entire 16-second tune by instrument and hand position)

Supplementary Material for Chapter 4.2.2

Table S9

Instrument Position	N	Non-standardized Coefficients		Standardized Coefficients	Sig.	R² Model
		Regression Coefficient B	Standard Error	Beta		
**6th Hand Position, Shoulder Protraction ("Coord x")**						
**A1**	60	21.776	0.753			0.711
**A2**	60	-7.746	1.065	-0.376	p<0.001	
**B1**	60	-13.154	1.065	-0.639	p<0.001	
**B2**	60	-17.050	1.065	-0.828	p<0.001	
**Free**	60	-12.638	1.065	-0.614	p<0.001	
**6th Hand Position, Shoulder Elevation ("Coord y")**						
**A1**	60	5.763	0.392			0.456
**A2**	60	-1.088	0.554	-0.129	n.s	
**B1**	60	-3.367	0.554	-0.398	p<0.001	
**B2**	60	-3.468	0.554	-0.410	p<0.001	
**Free**	60	-0.022	0.554	-0.003	n.s.	
**2nd Hand Position, Shoulder Protraction ("Coord x")**						
**A1**	30	13.047	0.884			0.400
**A2**	30	-4.914	1.250	-0.320	p<0.001	
**B1**	30	-9.181	1.250	-0.598	p<0.001	
**B2**	30	-11.283	1.250	-0.735	p<0.001	
**Free**	30	-5.347	1.250	-0.348	p<0.001	
**2nd Hand Position, Shoulder Elevation ("Coord y")**						
**A1**	30	3.475	0.492			0.189
**A2**	30	-0.947	0.696	-0.129	n.s.	
**B1**	30	-1.808	0.696	-0.246	p<0.05	
**B2**	30	-1.725	0.696	-0.234	p<0.05	
**Free**	30	1.631	0.696	0.222	p<0.05	

Table I: Regression analysis for shoulder protraction ("Coord x") and shoulder elevation ("Coord y") by instrument and hand position

Table S10

Multiple Comparison of Means (Scheffé) for Shoulder Protraction (“Coord x”), 2nd Hand Position						
Instrument Position		Mean Difference	Standard Error	Sig.	95% CI	
					Lower Bound	Upper Bound
1 A1	2 A2	4.914	1.250	p<0.01	1.014	8.814
	3 B1	9.181	1.250	p<0.001	5.281	13.080
	4 B2	11.283	1.250	p<0.001	7.384	15.183
	5 Free	5.347	1.250	p<0.01	1.448	9.247
2 A2	1 A1	-4.91389	1.250	p<0.01	-8.814	-1.014
	3 B1	4.267	1.250	p<0.05	0.367	8.166
	4 B2	6.369	1.250	p<0.001	2.470	10.269
	5 Free	0.433	1.250	n.s.	-3.466	4.333
3 B1	1 A1	-9.181	1.250	p<0.001	-13.080	-5.281
	2 A2	-4.267	1.250	p<0.05	-8.166	-0.367
	4 B2	2.103	1.250	n.s.	-1.797	6.002
	5 Free	-3.833	1.250	n.s.	-7.733	0.066
4 B2	1 A1	-11.283	1.250	p<0.001	-15.183	-7.384
	2 A2	-6.369	1.250	p<0.001	-10.269	-2.470
	3 B1	-2.103	1.250	n.s.	-6.002	1.797
	5 Free	-5.936	1.250	p<0.001	-9.836	-2.036
5 Free	1 A1	-5.347	1.250	p<0.01	-9.247	-1.448
	2 A2	-0.433	1.250	n.s.	-4.333	3.466
	3 B1	3.833	1.250	n.s.	-0.066	7.733
	4 B2	5.936	1.250	p<0.001	2.036	9.836

Multiple Comparison of Means (Scheffé) for Shoulder Protraction (“Coord x”), 6th Hand Position						
Instrument Position		Mean Difference	Standard Error	Sig.	95% CI	
					Lower Bound	Upper Bound
1 A1	2 A2	7.746	1.065	p<0.001	4.443	11.049
	3 B1	13.154	1.065	p<0.001	9.851	16.457
	4 B2	17.050	1.065	p<0.001	13.747	20.353
	5 Free	12.638	1.065	p<0.001	9.335	15.940
2 A2	1 A1	-7.746	1.065	p<0.001	-11.049	-4.443
	3 B1	5.408	1.065	p<0.001	2.105	8.711
	4 B2	9.304	1.065	p<0.001	6.001	12.607
	5 Free	4.892	1.065	p<0.001	1.589	8.195
3 B1	1 A1	-13.154	1.065	p<0.001	-16.457	-9.851
	2 A2	-5.408	1.065	p<0.001	-8.711	-2.105
	4 B2	3.896	1.065	p<0.05	0.593	7.199
	5 Free	-0.517	1.065	n.s.	-3.820	2.786
4 B2	1 A1	-17.050	1.065	p<0.001	-20.353	-13.747
	2 A2	-9.304	1.065	p<0.001	-12.607	-6.001
	3 B1	-3.896	1.065	p<0.05	-7.199	-0.593
	5 Free	-4.413	1.065	p<0.01	-7.715	-1.110
5 Free	1 A1	-12.638	1.065	p<0.001	-15.940	-9.335
	2 A2	-4.892	1.065	p<0.001	-8.195	-1.589
	3 B1	0.517	1.065	n.s.	-2.786	3.820
	4 B2	4.413	1.065	p<0.01	1.110	7.715

Multiple Comparison of Means (Scheffé) for Shoulder Elevation (“Coord y”), 2nd Hand Position						
Instrument Position		Mean Difference	Standard Error	Sig.	95% CI	
					Lower Bound	Upper Bound
1 A1	2 A2	0.947	0.696	0.763	-1.225	3.120
	3 B1	1.808	0.696	0.156	-0.364	3.981
	4 B2	1.725	0.696	0.195	-0.448	3.898
	5 Free	-1.631	0.696	0.247	-3.803	0.542
2 A2	1 A1	-0.947	0.696	0.763	-3.120	1.225
	3 B1	0.861	0.696	0.821	-1.311	3.034
	4 B2	0.778	0.696	0.870	-1.395	2.950
	5 Free	-2.578	0.696	p<0.05	-4.750	-0.405
3 B1	1 A1	-1.808	0.696	0.156	-3.981	0.364
	2 A2	-0.861	0.696	0.821	-3.034	1.311
	4 B2	-0.083	0.696	1.000	-2.256	2.089
	5 Free	-3.439	0.696	p<0.001	-5.611	-1.266
4 B2	1 A1	-1.725	0.696	0.195	-3.898	0.448
	2 A2	-0.778	0.696	0.870	-2.950	1.395
	3 B1	0.083	0.696	1.000	-2.089	2.256
	5 Free	-3.356	0.696	p<0.001	-5.528	-1.183
5 Free	1 A1	1.631	0.696	0.247	-0.542	3.803
	2 A2	2.578	0.696	p<0.05	0.405	4.750
	3 B1	3.439	0.696	p<0.001	1.266	5.611
	4 B2	3.356	0.696	p<0.001	1.183	5.528

Multiple Comparison of Means (Scheffé) for Shoulder Protraction (“Coord y”), 6th Hand Position						
Instrument Position		Mean Difference	Standard Error	Sig.	95% CI	
					Lower Bound	Upper Bound
1 A1	2 A2	1.088	0.554	n.s.	-0.631	2.806
	3 B1	3.367	0.554	p<0.001	1.648	5.085
	4 B2	3.468	0.554	p<0.001	1.749	5.187
	5 Free	0.022	0.554	n.s.	-1.697	1.741
2 A2	1 A1	-1.088	0.554	n.s.	-2.806	0.631
	3 B1	2.279	0.554	p<0.01	0.560	3.998
	4 B2	2.381	0.554	p<0.01	0.662	4.099
	5 Free	-1.065	0.554	n.s.	-2.784	0.653
3 B1	1 A1	-3.367	0.554	p<0.001	-5.085	-1.648
	2 A2	-2.279	0.554	p<0.01	-3.998	-0.560
	4 B2	0.101	0.554	n.s.	-1.617	1.820
	5 Free	-3.344	0.554	p<0.001	-5.063	-1.626
4 B2	1 A1	-3.468	0.554	p<0.001	-5.187	-1.749
	2 A2	-2.381	0.554	p<0.01	-4.099	-0.662
	3 B1	-0.101	0.554	n.s.	-1.820	1.617
	5 Free	-3.446	0.554	p<0.001	-5.165	-1.727
5 Free	1 A1	-0.022	0.554	n.s.	-1.741	1.697
	2 A2	1.065	0.554	n.s.	-0.653	2.784
	3 B1	3.344	0.554	p<0.001	1.626	5.063
	4 B2	3.446	0.554	p<0.001	1.727	5.165

Table J: Multiple comparison of means (Scheffé) for shoulder protraction (“Coord x”) and shoulder elevation (“Coord y”) by instrument and hand position

Supplementary Material for Chapter 4.3.1

Table S11

Time Segment	Violin Position	Non-standardized Coefficients: Regression Coefficient B	Non-standardized Coefficients: Standard Error	Standardized Coefficients: Beta	Sig.	R^2 model
**6th Hand Position (N=60 per Instrument Position, Fig 9)**						
**4th finger normal (segments "a", sec. 2.000-3.995 and 10.000-11.995)**	**A1**	26.262	0.335			0.393
	**A2**	-2.923	0.474	-0.354	p<0.001	
	**B1**	-2.029	0.474	-0.246	p<0.001	
	**B2**	-5.818	0.474	-0.704	p<0.001	
	**Free**	-4.906	0.474	-0.594	p<0.001	
**4th finger high (segment "b", sec. 14.000-15.995)**	**A1**	26.624	0.331			0.395
	**A2**	-3.085	0.469	-0.377	p<0.001	
	**B1**	-2.013	0.469	-0.246	p<0.001	
	**B2**	-5.813	0.469	-0.710	p<0.001	
	**Free**	-4.840	0.469	-0.592	p<0.001	
**All Hand Positions (N=90 per instrument position, dashed line - - - in Figs. 9 and 10)**						
**4th finger normal (segments "a")**	**A1**	25.326	0.335			0.329
	**A2**	-2.585	0.474	-0.268	p<0.001	
	**B1**	-2.835	0.474	-0.294	p<0.001	
	**B2**	-6.171	0.474	-0.639	p<0.001	
	**Free**	-5.470	0.474	-0.567	p<0.001	
**4th finger high (segment "b")**	**A1**	25.655	0.331			0.324
	**A2**	-2.737	0.468	-0.288	p<0.001	
	**B1**	-2.700	0.468	-0.284	p<0.001	
	**B2**	-6.088	0.468	-0.640	p<0.001	
	**Free**	-5.334	0.468	-0.561	p<0.001	
**2nd Hand Position (N=30 per instrument position, Fig. 10)**						
**4th finger normal (segments "a")**	**A1**	23.455	0.490			0.506
	**A2**	-1.908	0.693	-0.203	p<0.01	
	**B1**	-4.447	0.693	-0.474	p<0.001	
	**B2**	-6.877	0.693	-0.733	p<0.001	
	**Free**	-6.598	0.693	-0.703	p<0.001	
**4th finger high (segment "b")**	**A1**	23.717	0.493			0.476
	**A2**	-2.041	0.697	-0.223	p<0.01	
	**B1**	-4.072	0.697	-0.444	p<0.001	
	**B2**	-6.637	0.697	-0.724	p<0.001	
	**Free**	-6.321	0.697	-0.690	p<0.001	

Table K: Multiple regression analyses for “Reference Angle α” and the specific moments when the 4th fingers are involved in playing

Table S12

Multiple Comparisons of Means (Scheffé) for Reference Angle α: Normal and High 4th Finger, All Hand Positions United (N=450)

Time Segment	Instrument Position		Mean Difference	Standard Error	Sig.	95% CI Lower Bound	95% CI Upper Bound
4th finger normal (segments "a", sec. 2.000-3.995 and 10.000-11.995)	1 A1	2 A2	2.585	0.474	p<0.001	1.118	4.051
		3 B1	2.835	0.474	p<0.001	1.368	4.302
		4 B2	6.171	0.474	p<0.001	4.704	7.638
		5 Free	5.470	0.474	p<0.001	4.003	6.937
	2 A2	1 A1	-2.585	0.474	p<0.001	-4.051	-1.118
		3 B1	0.251	0.474	n.s.	-1.216	1.717
		4 B2	3.586	0.474	p<0.001	2.119	5.053
		5 Free	2.885	0.474	p<0.001	1.419	4.352
	3 B1	1 A1	-2.835	0.474	p<0.001	-4.302	-1.368
		2 A2	-0.251	0.474	n.s.	-1.717	1.216
		4 B2	3.335	0.474	p<0.001	1.869	4.802
		5 Free	2.635	0.474	p<0.001	1.168	4.102
	4 B2	1 A1	-6.171	0.474	p<0.001	-7.638	-4.704
		2 A2	-3.586	0.474	p<0.001	-5.053	-2.119
		3 B1	-3.335	0.474	p<0.001	-4.802	-1.869
		5 Free	-0.701	0.474	n.s.	-2.167	0.766
	5 Free	1 A1	-5.470	0.474	p<0.001	-6.937	-4.003
		2 A2	-2.885	0.474	p<0.001	-4.352	-1.419
		3 B1	-2.635	0.474	p<0.001	-4.102	-1.168
		4 B2	0.701	0.474	n.s.	-0.766	2.167
4th finger high (segment "b", sec. 14.000-15.995)	1 A1	2 A2	2.737	0.468	p<0.001	1.288	4.186
		3 B1	2.700	0.468	p<0.001	1.251	4.149
		4 B2	6.088	0.468	p<0.001	4.639	7.537
		5 Free	5.334	0.468	p<0.001	3.885	6.783
	2 A2	1 A1	-2.737	0.468	p<0.001	-4.186	-1.288
		3 B1	-0.037	0.468	n.s.	-1.486	1.412
		4 B2	3.351	0.468	p<0.001	1.902	4.800
		5 Free	2.597	0.468	p<0.001	1.148	4.046
	3 B1	1 A1	-2.700	0.468	p<0.001	-4.149	-1.251
		2 A2	0.037	0.468	n.s.	-1.412	1.486
		4 B2	3.388	0.468	p<0.001	1.939	4.837
		5 Free	2.634	0.468	p<0.001	1.185	4.083
	4 B2	1 A1	-6.088	0.468	p<0.001	-7.537	-4.639
		2 A2	-3.351	0.468	p<0.001	-4.800	-1.902
		3 B1	-3.388	0.468	p<0.001	-4.837	-1.939
		5 Free	-0.754	0.468	n.s.	-2.203	0.695
	5 Free	1 A1	-5.334	0.468	p<0.001	-6.783	-3.885
		2 A2	-2.597	0.468	p<0.001	-4.046	-1.148
		3 B1	-2.634	0.468	p<0.001	-4.083	-1.185
		4 B2	0.754	0.468	n.s.	-0.695	2.203

Multiple Comparisons of Means (Scheffé) for Reference Angle α: Normal and High 4th Finger for 2nd Hand Position (N=150)

Time Segment	Instrument Position		Mean Difference	Standard Error	Sig.	95% CI Lower Bound	95% CI Upper Bound
4th finger normal (segments "a", sec. 2.000-3.995 and 10.000-11.995)	1 A1	2 A2	1.908	0.693	n.s.	-0.254	4.069
		3 B1	4.447	0.693	p<0.001	2.286	6.608
		4 B2	6.877	0.693	p<0.001	4.716	9.038
		5 Free	6.598	0.693	p<0.001	4.436	8.759
	2 A2	1 A1	-1.908	0.693	n.s.	-4.069	0.254
		3 B1	2.539	0.693	p<0.05	0.378	4.701
		4 B2	4.969	0.693	p<0.001	2.808	7.130
		5 Free	4.690	0.693	p<0.001	2.528	6.851
	3 B1	1 A1	-4.447	0.693	p<0.001	-6.608	-2.286
		2 A2	-2.539	0.693	p<0.05	-4.701	-0.378
		4 B2	2.430	0.693	p<0.05	0.269	4.591
		5 Free	2.151	0.693	n.s.	-0.011	4.312
	4 B2	1 A1	-6.877	0.693	p<0.001	-9.038	-4.716
		2 A2	-4.969	0.693	p<0.001	-7.130	-2.808
		3 B1	-2.430	0.693	p<0.05	-4.591	-0.269
		5 Free	-0.279	0.693	n.s.	-2.441	1.882
	5 Free	1 A1	-6.598	0.693	p<0.001	-8.759	-4.436
		2 A2	-4.690	0.693	p<0.001	-6.851	-2.528
		3 B1	-2.151	0.693	n.s	-4.312	0.011
		4 B2	0.279	0.693	n.s.	-1.882	2.441
4th finger high (segment "b", sec. 14.000-15.995)	1 A1	2 A2	2.041	0.697	n.s.	-0.133	4.216
		3 B1	4.072	0.697	p<0.001	1.898	6.247
		4 B2	6.637	0.697	p<0.001	4.462	8.812
		5 Free	6.321	0.697	p<0.001	4.146	8.495
	2 A2	1 A1	-2.041	0.697	n.s.	-4.216	0.133
		3 B1	2.031	0.697	n.s.	-0.144	4.206
		4 B2	4.596	0.697	p<0.001	2.421	6.770
		5 Free	4.279	0.697	p<0.001	2.105	6.454
	3 B1	1 A1	-4.072	0.697	p<0.001	-6.247	-1.898
		2 A2	-2.031	0.697	n.s.	-4.206	0.144
		4 B2	2.565	0.697	p<0.05	0.390	4.739
		5 Free	2.248	0.697	p<0.05	0.074	4.423
	4 B2	1 A1	-6.637	0.697	p<0.001	-8.812	-4.462
		2 A2	-4.596	0.697	p<0.001	-6.770	-2.421
		3 B1	-2.565	0.697	p<0.05	-4.739	-0.390
		5 Free	-0.316	0.697	n.s.	-2.491	1.858
	5 Free	1 A1	-6.321	0.697	p<0.001	-8.495	-4.146
		2 A2	-4.279	0.697	p<0.001	-6.454	-2.105
		3 B1	-2.248	0.697	p<0.05	-4.423	-0.074
		4 B2	0.316	0.697	n.s.	-1.858	2.491

Multiple Comparisons of Means (Scheffé) for Reference Angle α: Normal and High 4th Finger for 6th Hand Position (N=300)

Time Segment	Instrument Position		Mean Difference	Standard Error	Sig.	95% CI Lower Bound	95% CI Upper Bound
4th finger normal (segments "a", sec. 2.000-3.995 and 10.000-11.995)	1 A1	2 A2	2.923	0.474	p<0.001	1.454	4.392
		3 B1	2.029	0.474	p<0.01	0.560	3.499
		4 B2	5.818	0.474	p<0.001	4.348	7.287
		5 Free	4.906	0.474	p<0.001	3.437	6.376
	2 A2	1 A1	-2.923	0.474	p<0.001	-4.392	-1.454
		3 B1	-0.894	0.474	n.s.	-2.363	0.576
		4 B2	2.895	0.474	p<0.001	1.425	4.364
		5 Free	1.983	0.474	p<0.01	0.514	3.453
	3 B1	1 A1	-2.029	0.474	p<0.01	-3.499	-0.560
		2 A2	0.894	0.474	n.s.	-0.576	2.363
		4 B2	3.788	0.474	p<0.001	2.319	5.257
		5 Free	2.877	0.474	p<0.001	1.408	4.346
	4 B2	1 A1	-5.818	0.474	p<0.001	-7.287	-4.348
		2 A2	-2.895	0.474	p<0.001	-4.364	-1.425
		3 B1	-3.788	0.474	p<0.001	-5.257	-2.319
		5 Free	-0.911	0.474	n.s.	-2.380	0.558
	5 Free	1 A1	-4.906	0.474	p<0.001	-6.376	-3.437
		2 A2	-1.983	0.474	p<0.01	-3.453	-0.514
		3 B1	-2.877	0.474	p<0.001	-4.346	-1.408
		4 B2	0.911	0.474	n.s.	-0.558	2.380
4th finger high (segment "b", sec. 14.000-15.995)	1 A1	2 A2	3.085	0.469	p<0.001	1.632	4.537
		3 B1	2.013	0.469	p<0.01	0.561	3.466
		4 B2	5.813	0.469	p<0.001	4.360	7.266
		5 Free	4.840	0.469	p<0.001	3.388	6.293
	2 A2	1 A1	-3.085	0.469	p<0.001	-4.537	-1.632
		3 B1	-1.071	0.469	n.s.	-2.524	0.381
		4 B2	2.728	0.469	p<0.001	1.276	4.181
		5 Free	1.756	0.469	p<0.01	0.303	3.208
	3 B1	1 A1	-2.013	0.469	p<0.01	-3.466	-0.561
		2 A2	1.071	0.469	n.s.	-0.381	2.524
		4 B2	3.800	0.469	p<0.001	2.347	5.253
		5 Free	2.827	0.469	p<0.001	1.374	4.280
	4 B2	1 A1	-5.813	0.469	p<0.001	-7.266	-4.360
		2 A2	-2.728	0.469	p<0.001	-4.181	-1.276
		3 B1	-3.800	0.469	p<0.001	-5.253	-2.347
		5 Free	-0.973	0.469	n.s.	-2.426	0.480
	5 Free	1 A1	-4.840	0.469	p<0.001	-6.293	-3.388
		2 A2	-1.756	0.469	p<0.01	-3.208	-0.303
		3 B1	-2.827	0.469	p<0.001	-4.280	-1.374
		4 B2	0.973	0.469	n.s.	-0.480	2.426

Table L: Multiple comparison of means (Scheffé) for "Reference Angle α" by instrument and hand position for the specific points in time when the normal and high 4th finger are played.

Supplementary Material for Chapter 4.3.2

Table S13

Instrument Position	Shoulder Motion	Point in Time	6th Hand Position N	Mean	Min.	Max.	SD	2nd Hand Position N	Mean	Min.	Max.	SD
All Positions United	Shoulder Protraction ("Coord x")	**Overall Average**	1200	11.66	-4.0	43.0	6.417	600	6.90	-6.0	32.0	4.106
	Shoulder Elevation ("Coord y")	**Overall Average**	1200	4.17	-3.0	20.0	1.651	600	2.91	-3.5	17.5	1.758
A1	x	**Beginning of tune** (sec. 0.000)	60	17.63	4.8	34.8	7.630	30	11.33	3.5	28.0	5.860
		**4th finger normal** (sec. 2.000 & 10.000 aggregated)	120	21.18	5.4	37.3	7.293	60	13.21	3.5	29.5	6.486
		**4th finger high** (sec. 14.000)	60	26.52	9.0	42.8	8.222	30	14.60	3.5	32.0	6.672
	y	Beginning of tune	60	4.87	0.3	11.8	2.701	30	3.17	-0.5	17.0	3.203
		4th finger normal	120	5.84	-1.1	13.8	3.225	60	3.46	-0.3	17.0	3.150
		4th finger high	60	6.58	-0.5	17.0	3.678	30	3.80	0.5	17.5	3.309
A2	x	Beginning of tune	60	11.80	3.5	25.3	5.634	30	7.35	-1.5	17.5	4.036
		4th finger normal	120	13.59	3.8	26.0	5.657	60	8.22	-1.0	18.5	4.341
		4th finger high	60	16.70	4.8	28.8	6.178	30	8.83	-0.5	20.0	4.582
	y	Beginning of tune	60	4.10	0.0	10.8	2.528	30	2.55	-0.5	6.0	1.864
		4th finger normal	120	4.82	0.4	10.8	2.641	60	2.53	0.0	6.0	1.745
		4th finger high	60	5.11	1.0	11.0	2.746	30	2.50	-0.5	6.5	1.808
B1	x	Beginning of tune	60	6.80	0.5	18.0	4.310	30	3.15	-2.5	9.5	2.746
		4th finger normal	120	7.98	0.4	17.8	4.195	60	3.88	-2.5	11.3	3.076
		4th finger high	60	11.08	2.0	23.0	5.456	30	4.57	-2.5	12.0	3.550
	y	Beginning of tune	60	2.23	-0.8	5.8	1.822	30	1.85	-2.5	6.5	1.950
		4th finger normal	120	2.42	-1.3	7.4	2.283	60	1.58	-3.5	6.3	2.154
		4th finger high	60	2.54	-3.0	7.8	2.539	30	1.57	-3.5	5.5	2.069
B2	x	Beginning of tune	60	3.69	-0.8	12.8	3.045	30	0.92	-6.0	7.5	2.823
		4th finger normal	120	4.60	-0.5	16.6	3.747	60	1.89	-4.5	9.5	3.009
		4th finger high	60	5.88	0.0	18.5	4.077	30	2.48	-4.5	9.5	3.062
	y	Beginning of tune	60	2.66	-0.5	20.0	3.536	30	2.12	-1.5	7.0	2.179
		4th finger normal	120	2.08	-0.8	5.8	1.594	60	1.60	-2.0	7.0	2.444
		4th finger high	60	2.15	-1.3	6.0	1.762	30	1.53	-2.0	7.5	2.637
Free	x	Beginning of tune	60	8.66	-4.0	26.0	6.710	30	7.98	-4.0	22.0	6.384
		4th finger normal	120	8.84	-2.9	24.0	6.670	60	7.73	-5.3	22.0	6.269
		4th finger high	60	9.92	-2.5	26.0	7.102	30	7.38	-6.0	22.5	6.446
	y	Beginning of tune	60	5.94	-1.3	18.0	5.008	30	5.42	-2.0	14.5	3.789
		4th finger normal	120	5.80	-0.8	17.5	4.418	60	5.07	-2.5	14.0	3.644
		4th finger high	60	5.48	-1.3	17.0	4.366	30	4.83	-2.5	13.0	3.378

Table M: Statistics for shoulder compensation movements by instrument and hand position when playing the normal and high 4th fingers of the tune. The table allows for comparison of 4th finger data with the shoulder position at the beginning of the tune and the overall mean for shoulder motion (grey rows at top of the table). For 6th hand position, shoulder protraction ("x"-values) shows a characteristic increase: lowest in the beginning of the tune and highest at second 14.000. For shoulder elevation ("y"-values), the increase of values applies to instrument positions A1, A2 and B1, but for instrument position B2 and Free either a decrease (Free) or other sequence (B2) is observed. For 2nd position, instrument position A1 is the only position where an increase of "y"-values (shoulder elevation) is observed. For all other instrument positions, the degree of shoulder elevation is highest at the beginning of the tune and decreases towards the end. For shoulder protraction ("Coord x") by instrument and hand position in the specific moments when the 4th fingers are used, multiple regression analysis shows highly significant differences between violin position A1 and all other instrument positions. This applies both to the normal 4th finger in 6th hand position (N=300, $p<.001$, r^2=505) and the normal 4th finger in 2nd hand position (N=150, $p<.001$, r^2=0.392). Comparable results are seen for the high 4th finger in 6th hand position (N=300, $p<.001$, r^2=557) and the high 4th finger in 2nd hand position (N=150, $p<.001$, r^2=0.400).

Table S14

Instrument Position	N	Non-standardized Coefficients: Regression Coefficient B	Standard Error	Standardized Coefficients: Beta	Sig.	R^2 Model
**4th Finger Normal, 2nd Position, Shoulder Protraction ("Coord x")**						
**A1**	30	13.208	0.905			0.392
**A2**	30	-4.992	1.279	-0.320	p<0.001	
**B1**	30	-9.325	1.279	-0.597	p<0.001	
**B2**	30	-11.317	1.279	-0.725	p<0.001	
**Free**	30	-5.475	1.279	-0.351	p<0.001	
**4th Finger Normal, 2nd Position, Shoulder Elevation ("Coord y")**						
**A1**	30	3.458	0.504			0.188
**A2**	30	-0.925	0.713	-0.123	n.s.	
**B1**	30	-1.875	0.713	-0.249	p<0.01	
**B2**	30	-1.858	0.713	-0.247	p<0.01	
**Free**	30	1.608	0.713	0.213	p<0.05	
**4th Finger Normal, 6th Position, Shoulder Protraction ("Coord x")**						
**A1**	60	21.179	0.739			0.505
**A2**	60	-7.588	1.046	-0.376	p<0.001	
**B1**	60	-13.196	1.046	-0.654	p<0.001	
**B2**	60	-16.575	1.046	-0.821	p<0.001	
**Free**	60	-12.338	1.046	-0.611	p<0.001	
**4th Finger Normal, 6th Position, Shoulder Elevation ("Coord y")**						
**A1**	60	5.838	0.389			0.229
**A2**	60	-1.021	0.550	-0.120	n.s.	
**B1**	60	-3.417	0.550	-0.402	p<0.001	
**B2**	60	-3.763	0.550	-0.442	p<0.001	
**Free**	60	-0.042	0.550	-0.005	n.s.	
**4th Finger High, 2nd Position, Shoulder Protraction ("Coord x")**						
**A1**	30	14.600	0.943			0.400
**A2**	30	-5.767	1.334	-0.352	p<0.001	
**B1**	30	-10.033	1.334	-0.612	p<0.001	
**B2**	30	-12.117	1.334	-0.739	p<0.001	
**Free**	30	-7.217	1.334	-0.440	p<0.001	
**4th Finger High, 2nd Position, Shoulder Elevation ("Coord y")**						
**A1**	30	3.800	0.504			0.185
**A2**	30	-1.300	0.713	-0.173	n.s.	
**B1**	30	-2.233	0.713	-0.297	p<0.01	
**B2**	30	-2.267	0.713	-0.302	p<0.01	
**Free**	30	1.033	0.713	0.137	n.s.	
**4th Finger High, 6th Position, Shoulder Protraction ("Coord x")**						
**A1**	60	26.517	0.829			0.557
**A2**	60	-9.817	1.172	-0.410	p<0.001	
**B1**	60	-15.433	1.172	-0.645	p<0.001	
**B2**	60	-20.633	1.172	-0.863	p<0.001	
**Free**	60	-16.600	1.172	-0.694	p<0.001	
**4th Finger High, 6th Position, Shoulder Elevation ("Coord y")**						
**A1**	60	6.583	0.410			0.231
**A2**	60	-1.475	0.580	-0.164	p<0.01	
**B1**	60	-4.042	0.580	-0.450	p<0.001	
**B2**	60	-4.433	0.580	-0.493	p<0.001	
**Free**	60	-1.100	0.580	-0.122	n.s.	

Table N: Multiple regression analyses for shoulder protraction ("Coord x") and ("Coord y") by instrument and hand position for the specific moments when the 4th fingers are involved in playing. Regression analysis for

shoulder elevation (“Coord y”) by instrument and hand position shows a wider spectrum of significance levels: Highly significant differences are reported for the 4th finger normal in 6th hand position between A1 and B1 as well as B2 (N=300, $p<.001$, $r^2=.229$) and for the high 4th finger in 6th position between A1 and B1 as well as B2 (N=300, $p<.001$, $r^2=.231$). The next-highest significance levels are reached by the normal 4th finger in 2nd hand position with A1 differing highly significantly from B1 and B2 (N=150, $p<.010$, $r^2=0.188$) and significantly from Free (N=150, $p<.05$, $r^2=0.188$). For the high 4th finger in 2nd hand positions, differences between A1 and B1 and B2 are reported as highly significant (N=300, $p<.010$, $r^2=.185$) and for the high 4th finger in 6th hand position, position A1 differs significantly from A2 (N=300, $p<.05$, $r^2=.231$). For further details, please consult Table N in supplementary material. Results suggest that significance levels are higher when looking at shoulder protraction when the 4th fingers are played. Also, r^2model and therefore variance of shoulder protraction within a given hand and instrument position when the normal or high 4thfingers are played is higher than r^2 model for shoulder elevation.

Table S15

Multiple Comparisons of Means (Scheffé) by Instrument Position for Shoulder Protraction ("Coord x"), 4th Finger Normal, 2nd Hand Position

Instrument Position		Mean Difference	Standard Error	Sig.	95% CI	
					Lower Bound	Upper Bound
1 A1	2 A2	4.99167	1.279	p<0.01	0.999	8.984
	3 B1	9.325	1.279	p<0.001	5.333	13.317
	4 B2	11.31667	1.279	p<0.001	7.324	15.309
	5 Free	5.475	1.279	p<0.01	1.483	9.467
2 A2	1 A1	-4.99167	1.279	p<0.01	-8.984	-0.999
	3 B1	4.33333	1.279	p<0.05	0.341	8.326
	4 B2	6.325	1.279	p<0.001	2.333	10.317
	5 Free	0.48333	1.279	n.s.	-3.509	4.476
3 B1	1 A1	-9.325	1.279	p<0.001	-13.317	-5.333
	2 A2	-4.33333	1.279	p<0.05	-8.326	-0.341
	4 B2	1.99167	1.279	n.s.	-2.001	5.984
	5 Free	-3.85000	1.279	n.s.	-7.842	0.142
4 B2	1 A1	-11.31667	1.279	p<0.001	-15.309	-7.324
	2 A2	-6.325	1.279	p<0.001	-10.317	-2.333
	3 B1	-1.99167	1.279	n.s.	-5.984	2.001
	5 Free	-5.84167	1.279	p<0.01	-9.834	-1.849
5 Free	1 A1	-5.475	1.279	p<0.01	-9.467	-1.483
	2 A2	-0.48333	1.279	n.s.	-4.476	3.509
	3 B1	3.85000	1.279	n.s.	-0.142	7.842
	4 B2	5.84167	1.279	p<0.01	1.849	9.834

Multiple Comparisons of Means (Scheffé) by Instrument Position for Shoulder Elevation ("Coord y"), 4th Finger Normal, 2nd Hand Position

Instrument Position		Mean Difference	Standard Error	Sig.	95% CI	
					Lower Bound	Upper Bound
1 A1	2 A2	0.925	0.713	n.s.	-1.300	3.150
	3 B1	1.875	0.713	n.s.	-0.350	4.100
	4 B2	1.858	0.713	n.s.	-0.366	4.083
	5 Free	-1.608	0.713	n.s.	-3.833	0.616
2 A2	1 A1	-0.925	0.713	n.s.	-3.150	1.300
	3 B1	0.950	0.713	n.s.	-1.275	3.175
	4 B2	0.933	0.713	n.s.	-1.291	3.158
	5 Free	-2.533	0.713	p<0.05	-4.758	-0.309
3 B1	1 A1	-1.875	0.713	n.s.	-4.100	0.350
	2 A2	-0.950	0.713	n.s.	-3.175	1.275
	4 B2	-0.017	0.713	n.s.	-2.241	2.208
	5 Free	-3.483	0.713	p<0.001	-5.708	-1.259
4 B2	1 A1	-1.858	0.713	n.s.	-4.083	0.366
	2 A2	-0.933	0.713	n.s.	-3.158	1.291
	3 B1	0.017	0.713	n.s.	-2.208	2.241
	5 Free	-3.467	0.713	p<0.001	-5.691	-1.242
5 Free	1 A1	1.608	0.713	n.s.	-0.616	3.833
	2 A2	2.533	0.713	p<0.05	0.309	4.758
	3 B1	3.483	0.713	p<0.001	1.259	5.708
	4 B2	3.467	0.713	p<0.001	1.242	5.691

Multiple Comparisons of Means (Scheffé) by Instrument Position for Shoulder Protraction ("Coord x"), 4th Finger Normal, 6th Hand Position

Instrument Position		Mean Difference	Standard Error	Sig.	95% CI	
					Lower Bound	Upper Bound
1 A1	2 A2	7.588	1.046	p<0.001	4.346	10.829
	3 B1	13.196	1.046	p<0.001	9.955	16.437
	4 B2	16.575	1.046	p<0.001	13.334	19.816
	5 Free	12.338	1.046	p<0.001	9.096	15.579
2 A2	1 A1	-7.588	1.046	p<0.001	-10.829	-4.346
	3 B1	5.608	1.046	p<0.001	2.367	8.850
	4 B2	8.988	1.046	p<0.001	5.746	12.229
	5 Free	4.750	1.046	p<0.001	1.509	7.991
3 B1	1 A1	-13.196	1.046	p<0.001	-16.437	-9.955
	2 A2	-5.608	1.046	p<0.001	-8.850	-2.367
	4 B2	3.379	1.046	p<0.05	0.138	6.620
	5 Free	-0.858	1.046	n.s.	-4.100	2.383
4 B2	1 A1	-16.575	1.046	p<0.001	-19.816	-13.334
	2 A2	-8.988	1.046	p<0.001	-12.229	-5.746
	3 B1	-3.379	1.046	p<0.05	-6.620	-0.138
	5 Free	-4.238	1.046	p<0.01	-7.479	-0.996
5 Free	1 A1	-12.338	1.046	p<0.001	-15.579	-9.096
	2 A2	-4.750	1.046	p<0.001	-7.991	-1.509
	3 B1	0.858	1.046	n.s.	-2.383	4.100
	4 B2	4.238	1.046	p<0.01	0.996	7.479

Multiple Comparisons of Means (Scheffé) by Instrument Position for Shoulder Elevation ("Coord y"), 4th Finger Normal, 6th Hand Position

Instrument Position		Mean Difference	Standard Error	Sig.	95% CI	
					Lower Bound	Upper Bound
1 A1	2 A2	1.021	0.550	n.s.	-0.684	2.726
	3 B1	3.417	0.550	p<0.001	1.712	5.122
	4 B2	3.763	0.550	p<0.001	2.057	5.468
	5 Free	0.042	0.550	n.s.	-1.663	1.747
2 A2	1 A1	-1.021	0.550	n.s.	-2.726	0.684
	3 B1	2.396	0.550	p<0.01	0.691	4.101
	4 B2	2.742	0.550	p<0.001	1.037	4.447
	5 Free	-0.979	0.550	n.s.	-2.684	0.726
3 B1	1 A1	-3.417	0.550	p<0.001	-5.122	-1.712
	2 A2	-2.396	0.550	p<0.01	-4.101	-0.691
	4 B2	0.346	0.550	n.s.	-1.359	2.051
	5 Free	-3.375	0.550	p<0.001	-5.080	-1.670
4 B2	1 A1	-3.763	0.550	p<0.001	-5.468	-2.057
	2 A2	-2.742	0.550	p<0.001	-4.447	-1.037
	3 B1	-0.346	0.550	n.s.	-2.051	1.359
	5 Free	-3.721	0.550	p<0.001	-5.426	-2.016
5 Free	1 A1	-0.042	0.550	n.s.	-1.747	1.663
	2 A2	0.979	0.550	n.s.	-0.726	2.684
	3 B1	3.375	0.550	p<0.001	1.670	5.080
	4 B2	3.721	0.550	p<0.001	2.016	5.426

Multiple Comparisons of Means (Scheffé) by Instrument Position for Shoulder Protraction ("Coord x"), 4th Finger High, 2nd Hand Position

Instrument Position		Mean Difference	Standard Error	Sig.	95% CI	
					Lower Bound	Upper Bound
1 A1	2 A2	5.76667	1.334	p<0.01	1.605	9.928
	3 B1	10.03333	1.334	p<0.001	5.872	14.195
	4 B2	12.11667	1.334	p<0.001	7.955	16.278
	5 Free	7.21667	1.334	p<0.001	3.055	11.378
2 A2	1 A1	-5.76667	1.334	p<0.01	-9.928	-1.605
	3 B1	4.26667	1.334	p<0.05	0.105	8.428
	4 B2	6.35	1.334	p<0.001	2.188	10.512
	5 Free	1.45000	1.334	n.s.	-2.712	5.612
3 B1	1 A1	-10.03333	1.334	p<0.001	-14.195	-5.872
	2 A2	-4.26667	1.334	p<0.05	-8.428	-0.105
	4 B2	2.08333	1.334	n.s.	-2.078	6.245
	5 Free	-2.81667	1.334	n.s.	-6.978	1.345
4 B2	1 A1	-12.11667	1.334	p<0.001	-16.278	-7.955
	2 A2	-6.35	1.334	p<0.001	-10.512	-2.188
	3 B1	-2.08333	1.334	n.s.	-6.245	2.078
	5 Free	-4.9	1.334	p<0.05	-9.062	-0.738
5 Free	1 A1	-7.21667	1.334	p<0.001	-11.378	-3.055
	2 A2	-1.45000	1.334	n.s.	-5.612	2.712
	3 B1	2.81667	1.334	n.s.	-1.345	6.978
	4 B2	4.9	1.334	p<0.05	0.738	9.062

Multiple Comparisons of Means (Scheffé) by Instrument Position for Shoulder Elevation ("Coord y"), 4th Finger High, 2nd Hand Position

Instrument Position		Mean Difference	Standard Error	Sig.	95% CI	
					Lower Bound	Upper Bound
1 A1	2 A2	1.300	0.713	n.s.	-0.924	3.524
	3 B1	2.233	0.713	p<0.05	0.009	4.458
	4 B2	2.267	0.713	p<0.05	0.042	4.491
	5 Frei	-1.033	0.713	n.s.	-3.258	1.191
2 A2	1 A1	-1.300	0.713	n.s.	-3.524	0.924
	3 B1	0.933	0.713	n.s.	-1.291	3.158
	4 B2	0.967	0.713	n.s.	-1.258	3.191
	5 Frei	-2.333	0.713	n.s.	-4.558	-0.109
3 B1	1 A1	-2.233	0.713	p<0.05	-4.458	-0.009
	2 A2	-0.933	0.713	n.s.	-3.158	1.291
	4 B2	0.033	0.713	n.s.	-2.191	2.258
	5 Frei	-3.267	0.713	p<0.01	-5.491	-1.042
4 B2	1 A1	-2.267	0.713	p<0.05	-4.491	-0.042
	2 A2	-0.967	0.713	n.s.	-3.191	1.258
	3 B1	-0.033	0.713	n.s.	-2.258	2.191
	5 Frei	-3.300	0.713	p<0.001	-5.524	-1.076
5 Frei	1 A1	1.033	0.713	n.s.	-1.191	3.258
	2 A2	2.333	0.713	p<0.05	0.109	4.558
	3 B1	3.267	0.713	p<0.01	1.042	5.491
	4 B2	3.300	0.713	p<0.001	1.076	5.524

Multiple Comparisons of Means (Scheffé) by Instrument Position for Shoulder Protraction ("Coord x"), 4th Finger High,6th Hand Position

Instrument Position		Mean Difference	Standard Error	Sig.	95% CI	
					Lower Bound	Upper Bound
1 A1	2 A2	9.817	1.172	p<0.001	6.184	13.449
	3 B1	15.433	1.172	p<0.001	11.801	19.066
	4 B2	20.633	1.172	p<0.001	17.001	24.266
	5 Free	16.600	1.172	p<0.001	12.968	20.232
2 A2	1 A1	-9.817	1.172	p<0.001	-13.449	-6.184
	3 B1	5.617	1.172	p<0.001	1.984	9.249
	4 B2	10.817	1.172	p<0.001	7.184	14.449
	5 Free	6.783	1.172	p<0.001	3.151	10.416
3 B1	1 A1	-15.433	1.172	p<0.001	-19.066	-11.801
	2 A2	-5.617	1.172	p<0.001	-9.249	-1.984
	4 B2	5.200	1.172	p<0.01	1.568	8.832
	5 Free	1.167	1.172	0.911	-2.466	4.799
4 B2	1 A1	-20.633	1.172	p<0.001	-24.266	-17.001
	2 A2	-10.817	1.172	p<0.001	-14.449	-7.184
	3 B1	-5.200	1.172	p<0.01	-8.832	-1.568
	5 Free	-4.033	1.172	p<0.05	-7.666	-0.401
5 Free	1 A1	-16.600	1.172	p<0.001	-20.232	-12.968
	2 A2	-6.783	1.172	p<0.001	-10.416	-3.151
	3 B1	-1.167	1.172	0.911	-4.799	2.466
	4 B2	4.033	1.172	p<0.05	0.401	7.666

Multiple Comparisons of Means (Scheffé) by Instrument Position for Shoulder Elevation ("Coord y"), 4th Finger High, 6th Hand Position

Instrument Position		Mean Difference	Standard Error	Sig.	95% CI	
					Lower Bound	Upper Bound
1 A1	2 A2	1.475	0.580	n.s.	-0.323	3.273
	3 B1	4.042	0.580	p<0.001	2.243	5.840
	4 B2	4.433	0.580	p<0.001	2.635	6.232
	5 Free	1.100	0.580	n.s.	-0.698	2.898
2 A2	1 A1	-1.475	0.580	n.s.	-3.273	0.323
	3 B1	2.567	0.580	p<0.01	0.768	4.365
	4 B2	2.958	0.580	p<0.001	1.160	4.757
	5 Free	-0.375	0.580	n.s.	-2.173	1.423
3 B1	1 A1	-4.042	0.580	p<0.001	-5.840	-2.243
	2 A2	-2.567	0.580	p<0.01	-4.365	-0.768
	4 B2	0.392	0.580	n.s.	-1.407	2.190
	5 Free	-2.942	0.580	p<0.001	-4.740	-1.143
4 B2	1 A1	-4.433	0.580	p<0.001	-6.232	-2.635
	2 A2	-2.958	0.580	p<0.001	-4.757	-1.160
	3 B1	-0.392	0.580	n.s.	-2.190	1.407
	5 Free	-3.333	0.580	p<0.001	-5.132	-1.535
5 Free	1 A1	-1.100	0.580	n.s.	-2.898	0.698
	2 A2	0.375	0.580	n.s.	-1.423	2.173
	3 B1	2.942	0.580	p<0.001	1.143	4.740
	4 B2	3.333	0.580	p<0.001	1.535	5.132

Table O: Multiple comparison of means (Scheffé) for shoulder protraction (“Coord x”) and shoulder elevation (“Coord y”) by instrument and hand position in the specific points in time when the 4th finger is played during the tune. A post-hoc, multiple comparison of means (Scheffé) for shoulder protraction (“Coord x”) and elevation (“Coord y”) regarding the specific time segments for the normal and high 4th fingers yields the highest number of statistically significant differences for shoulder protraction (“Coord x”) in 6th position: In this specific case, the 18 out 20 comparisons of means, both for the normal and the high 4th finger reach highest significance levels with the only non-significant result being the comparison between instrument position B1 and Free. For 2nd hand position, shoulder protraction (“Coord x”) also yields 14 out of 20 statistically significant results both for the normal and high 4th finger. However, the overall number of significant results (14 out of 20 for the normal and high 4th finger each) is reduced compared to results for 6th hand position. For shoulder elevation (“Coord y”), the number of statistically significant results further decreases to 12 out of 20 comparisons for both the normal and high 4th finger in 6th hand position and, finally, multiple comparisons of means for “Coord y” in 2ndposition yields the smallest number of statistically significant results, with 6 out of 20 comparisons being statistically significant for the normal 4th finger and 10 out of 20 comparisons for the high 4th finger. For a detailed overview, please consult Table O in the Supplemental Material.

Supplementary Material for Chapter 4.4.1

Table S16

		Shoulder Movement and Reference Angle α Correlations by Instrument Position, Hand Position and Time Segment (Pearson's *r*, Sig., 2-tailed)										
		Entire tune (sec. 0.000-15.995)										
	Shoulder Motion	Time Point	Angle α for A1		Angle α for A2		Angle α for B1		Angle α for B2		Angle α for Free	
			r	*p*	*r*	*p*	*r*	*p*	*r*	*p*	*r*	*p*
6th Hand Position	Shoulder Protraction ("Coord x"), N=60 per time point	Sec. 0.000 (beginning of tune)	0.201	0.124	0.304	p<0.05	0.086	n.s.	0.268	p<0.05	0.425	p<0.01
		Sec. 2.000 & 10.000_aggregated (4th finger normal)	0.166	0.204	0.238	n.s.	0.103	n.s.	0.123	n.s.	0.502	p<0.001
		Sec. 14.000 (4th finger high)	0.167	0.202	0.218	n.s.	0.011	n.s.	0.112	n.s.	0.529	p<0.001
	Shoulder Elevation ("Coord y"), N=60 per time point	Sec. 0.000 (beginning of tune)	0.146	0.265	0.225	n.s.	0.291	p<0.05	0.084	n.s.	0.299	p<0.05
		Sec. 2.000 & 10.000_aggregated (4th finger normal)	0.157	0.232	0.222	n.s.	0.232	n.s.	0.107	n.s.	0.260	p<0.05
		Sec. 14.000 (4th finger high)	-0.014	0.917	0.190	n.s.	0.199	n.s.	0.052	n.s.	0.245	0.059
2nd Hand Position	"Coord x", N=30	Sec. 0.000 (beginning of tune)	0.153	0.421	0.372	p<0.05	0.236	n.s.	0.171	n.s.	0.434	p<0.05
		Sec. 2.000 & 10.000_aggregated (4th finger normal)	0.156	0.409	0.360	n.s.	0.250	n.s.	0.092	n.s.	0.501	p<0.01
		Sec. 14.000 (4th finger high)	0.134	0.480	0.338	n.s.	0.298	0.110	0.069	n.s.	0.545	p<0.01
	"Coord y", N=30	Sec. 0.000 (beginning of tune)	0.052	0.785	-0.054	n.s.	0.111	n.s.	-0.071	n.s.	0.004	0.982
		Sec. 2.000 & 10.000_aggregated (4th finger normal)	0.048	0.801	0.056	n.s.	0.064	n.s.	-0.023	n.s.	-0.013	0.946
		Sec. 14.000 (4th finger high)	-0.051	0.788	0.022	n.s.	0.087	n.s.	-0.011	n.s.	0.038	0.841
		4th finger normal (Time segments "a", sec. 2.000-3.995 and 10.000-11.995)										
6th Hand Position	"Coord x", N=60	Sec. 2.000 & 10.000_aggregated (4th finger normal)	0.162	0.216	0.250	n.s.	0.099	n.s.	0.162	n.s.	0.509	p<0.001
	"Coord y", N=60	Sec. 2.000 & 10.000_aggregated (4th finger normal)	-0.018	0.890	0.167	n.s.	0.163	n.s.	0.000	n.s.	0.260	p<0.05
2nd Hand Position	Coord x, N=30	Sec. 2.000 & 10.000_aggregated (4th finger normal)	0.161	0.396	0.361	p<0.05	0.258	n.s.	0.123	n.s.	0.509	p<0.01
	Coord y, N=30	Sec. 2.000 & 10.000_aggregated (4th finger normal)	0.041	0.829	0.049	n.s.	0.040	n.s.	-0.061	n.s.	-0.006	0.974
		4th finger high (Time segment "b", sec. 14.000 - 15.995										
6th Hand Position	"Coord x", N=60	Sec. 14.000 (4th finger high)	0.206	0.115	0.223	n.s.	0.002	n.s.	0.131	n.s.	0.459	p<0.001
	"Coord y", N=60	Sec. 14.000 (4th finger high)	0.012	0.927	0.174	n.s.	0.156	n.s.	0.017	n.s.	0.286	p<0.05
2nd Hand Position	x, N=30	Sec. 14.000 (4th finger high)	0.172	0.362	0.363	p<0.05	0.324	n.s.	0.068	n.s.	0.534	p<0.01
	y, N=30	Sec. 14.000 (4th finger high)	-0.069	0.715	0.048	n.s.	0.042	n.s.	-0.035	n.s.	0.095	0.618

Table P: Detailed correlation analysis between shoulder protraction ("Coord x"), shoulder elevation ("Coord y") and "Reference Angle α" by instrument and hand position for the entire 16-second tune as well as the specific moments when the 4th fingers are played

Supplementary Material for Chapter 4.4.2

Table S17

		Reference Angle α and Biomechanics Correlations by Instrument Position, Hand Position and Time Segment																							
		Entire tune (sec. 0.000-15.995)								4th finger normal (Time segments "a", sec. 2.000-3.995 and 10.000-11.995)								4th finger high (Time segment "b", sec. 14.000 - 15.995							
		Diff_3_5		BM_Sup_pass_250		BM_Sup_pass_500		BM_Abd_Daumen		Diff_3_5		BM_Sup_pass_250		BM_Sup_pass_500		BM_Abd_Daumen		Diff_3_5		BM_Sup_pass_250		BM_Sup_pass_500		BM_Abd_Daumen	
Violin Position	Reference Angle α for Hand Position, N per observation	*r*	*p*	*r*	*p*	*r*	*p*	*r*	*p*	*r*	*p*	*r*	*p*	*r*	*p*	*r*	*p*	*r*	*p*	*r*	*p*	*r*	*p*	*r*	*p*
A1	6th Hand Position, N=60	0.028	0.832	-0.539	p<0.001	-0.558	p<0.001	0.297	p<0.05	0.019	0.887	-0.513	p<0.001	-0.537	p<0.001	0.327	p<0.05	0.014	0.917	-0.527	p<0.001	-0.528	p<0.001	0.347	p<0.01
	2nd Hand Position, N=30	-0.032	0.866	-0.475	p<0.01	-0.506	p<0.01	0.301	0.106	-0.042	0.825	-0.466	p<0.01	-0.501	p<0.01	0.301	0.106	-0.047	0.806	-0.434	p<0.05	-0.449	p<0.05	0.376	p<0.05
A2	6th Hand Position, N=60	0.042	0.753	-0.565	p<0.001	-0.587	p<0.001	0.288	p<0.05	0.037	0.778	-0.539	p<0.001	-0.575	p<0.001	0.310	p<0.05	0.030	0.818	-0.552	p<0.001	-0.561	p<0.001	0.331	p<0.01
	2nd Hand Position, N=30	-0.029	0.877	-0.531	p<0.01	-0.505	p<0.01	0.233	0.215	-0.027	0.888	-0.528	p<0.01	-0.506	p<0.01	0.242	0.198	-0.050	0.791	-0.487	p<0.01	-0.440	p<0.05	0.289	0.122
B1	6th Hand Position, N=60	0.016	0.903	-0.504	p<0.001	-0.497	p<0.001	0.256	p<0.05	-0.009	0.948	-0.470	p<0.001	-0.478	p<0.001	0.274	p<0.05	-0.026	0.846	-0.464	p<0.001	-0.473	p<0.001	0.303	p<0.05
	2nd Hand Position, N=30	-0.040	0.833	-0.410	p<0.05	-0.362	p<0.05	0.224	0.233	-0.071	0.708	-0.388	p<0.05	-0.351	p<0.05	0.218	0.248	-0.110	0.563	-0.316	0.089	-0.268	0.152	0.310	0.096
B2	6th Hand Position, N=60	0.045	0.734	-0.478	p<0.01	-0.502	p<0.001	0.299	p<0.05	0.036	0.786	-0.426	p<0.01	-0.477	p<0.001	0.340	p<0.001	0.017	0.897	-0.437	p<0.001	-0.447	p<0.001	0.339	p<0.01
	2nd Hand Position, N=30	0.027	0.889	-0.407	p<0.05	-0.413	p<0.05	0.204	0.280	0.003	0.986	-0.380	p<0.05	-0.394	p<0.05	0.227	0.228	-0.050	0.794	-0.302	0.105	-0.272	0.145	0.266	0.155
Free	6th Hand Position, N=60	0.097	0.461	-0.377	p<0.01	-0.433	p<0.01	0.041	0.754	0.061	0.645	-0.332	p<0.01	-0.391	p<0.01	0.092	0.483	0.082	0.535	-0.416	p<0.01	-0.442	p<0.001	0.104	0.427
	2nd Hand Position, N=30	0.097	0.611	-0.191	0.312	-0.283	0.130	-0.032	0.867	0.057	0.765	-0.206	0.276	-0.294	0.114	-0.016	0.934	0.021	0.914	-0.171	0.366	-0.228	0.226	0.057	0.765

Table Q: Correlation analysis between biomechanical parameters and elbow compensation movement (“Reference Angle α”) by instrument and hand position for the entire 16-second tune as well as the specific moments when the 4th fingers are played
